# *In situ* preparation of molybdenum-dioxide-incorporated carbonized silk fiber and its application in supercapacitors

**DOI:** 10.3389/fbioe.2022.1059399

**Published:** 2022-11-17

**Authors:** Yansong Ji, Xiaoning Zhang, Yong Zhu, Michael L. Norton, Lunfu Shen, Wenhui Tan, Xi Zheng, Shuo Li

**Affiliations:** ^1^ State Key Laboratory of Silkworm Genome Biology, College of Sericulture, Textile and Biomass Sciences, Southwest University, Chongqing, China; ^2^ Department of Chemistry, Marshall University, Huntington, WV, United States; ^3^ Chongqing Sericulture Science and Technology Research Institute, Chongqing Sericulture Technology Extension Station, Chongqing, China

**Keywords:** silkworms, modified mulberry leaves, carbonized silk, molybdenum dioxide, supercapacitors

## Abstract

A previous study found that the capacitive behavior of nanoparticles fed to the silkworm can be delivered to carbonized silk fibers, which can be used to fabricate electrodes for the construction of flexible supercapacitors. However, the tendency of nanoparticles to aggregate decreases the quantity of nanoparticles that enter the silk and therefore reduces the capacitance performance of the prepared carbonized silk. Here, we sprayed ammonium molybdate tetrahydrate (AMT) on the surface of mulberry leaves used for feeding silkworms and investigated the effect of feeding AMT on the growth of silkworms and the properties of spun silk. The precursor incorporated into the silk was converted into scattered MoO_2_ NPs, which were embedded within the carbonized silk fiber (CSF) *via* carbothermal reduction. The specific capacitance of CSF obtained from silkworms fed with an aqueous solution of AMT-treated mulberry leaves reached up to 298 F/g at 0.2 g/A, which is much higher than that of the control group (102 F/g). Since AMT is highly water-soluble, and its concentration can be easily modulated, we believe that the proposed strategy is feasible for the large-scale fabrication of CSF with enhanced capacitive performance.

## 1 Introduction

In recent years, the huge demand for wearable devices has promoted the rapid growth of flexible electronic components such as supercapacitors, which can serve as energy-storage devices in self-powered wearable systems (Wang et al., 2021). It is noteworthy that N doping can effectively enhance the capacitive performance of carbon-electrode supercapacitors (Wei et al., 2022). The carbonization of N-containing biomaterials is an effective strategy for the production of N-doped carbon (Feng et al., 2021), and much research has focused on the fabrication of N-doped carbon electrodes from the graphitization of natural silk (Yang et al., 2021; Zhou et al., 2022).

Such carbonized silk has been shown to exhibit high electrical conductivity and flexibility (Hou C. et al., 2021); therefore, it is a suitable candidate for use as an electrode material in flexible supercapacitors. Multiple strategies to improve the capacitance of carbonized silk have been reported (Li et al., 2019; Pan et al., 2020). A common strategy involves the incorporation of electrochemically active substances after preparing the carbonized silk (Li et al., 2019; Xia et al., 2021). Such a post functionalization method is likely to suffer from the detachment of the active material from the silk-derived carbon support, resulting in capacity-fading behavior during the charge and discharge cycles.

Feeding silkworms with electrochemically active nanomaterials is an alternative strategy for generating carbonized silk with an enhanced supercapacitive performance. In this process, the nanoparticles are incorporated into silk and retained in the encapsulated form after pyrolysis. Although this approach is sufficient to prevent detachment of these active particles from the carbonized silk, previous studies (Liang et al., 2020) have indicated that the aggregation of nanoparticles during feeding appears to compromise the nanoparticle characteristics crucial for their application in supercapacitors. In addition, aggregation results in a loss of uniformity in the active-substance distribution in the silk product because the nanoparticles cannot pass across the epithelial tissue of the silk gland when they aggregate into large clusters.

Zhuang et al. (2014) reported that molybdenum dioxide (MoO_2_) particles can be prepared *in situ* by the carbothermal reduction of ammonium molybdate tetrahydrate (AMT). Owing to its metal-like conductivity and environmental friendliness, MoO_2_ is a promising pseudocapacitive candidate and has been used extensively in supercapacitor-related devices (Zhang et al., 2020). We assume that AMT will uniformly disperse in silk because it can be evenly distributed over the mulberry leaf owing to its good solubility. Furthermore, because silkworm silk is constructed from oriented nanofibrils (Wang et al., 2020), the AMT incorporated within the silk can be converted into MoO_2_ nanoparticles after pyrolysis owing to its limited diffusion within the void spaces of the nanosized filaments.

In this study, the mechanically enhanced silk were obtained by feeding silkworm AMT-solution-sprayed mulberry leaves. In addition, a strategy for the *in situ* preparation of MoO_2_ NPs embedded in silk-derived carbon was explored. The composites exhibited enhanced capacitance. In comparison with feeding nanoparticles to silkworms, the proposed strategy effectively avoids the aggregation of nanoparticles. Therefore, the precursor amount can be modulated, allowing the electrochemically active substance to be more efficiently incorporate into the CSF. Additionally, metallic salts are less expensive than metallic nanoparticles, making them more practical for the large-scale fabrication and application of CSF.

## 2 Materials and methods

### 2.1 Materials and reagents

The silkworm strain used in the experiment was *Bombyx mori* (871 × 872), sourced from the Chongqing Sericulture Science and Technology Research Institute (Chongqing, China). Ammonium molybdate tetrahydrate (AMT) was purchased from Shanghai Darui Fine Chemical Co., Ltd. (Shanghai, China); sodium chloride was purchased from Shanghai Aladdin Bio-Chem Technology Co., Ltd. (Shanghai, China); nitric and perchloric acids were purchased from Beijing Institute of Chemical Reagents (Beijing, China); sodium carbonate and ethanol were purchased from Chongqing Chuandong Chemical Co., Ltd. (Chongqing, China); potassium bromide was purchased from Sangon Biotech Co., Ltd. (Shanghai, China); hematoxylin and eosin (H&E) staining kits were purchased from Nanchang Yulu Experimental Equipment Co., Ltd. (Nanchang, China); dimethylbenzene and poly (tetrafluoroethylene) were purchased from Shanghai Macklin Biochemical Co., Ltd. (Shanghai, China); Super P was purchased from Canrd New Energy Technology Co., Ltd. (Guangzhou, China); and foam Ni was purchased from Taiyuan Lizhiyuan Co., Ltd. (Shanxi, China). All chemicals were of analytical grade and used without further purification. The deionized (DI) water used in this work was produced using a Milli-Q Direct-8 purification system (resistivity >18 MΩ cm, Millipore Inc., France) onsite.

### 2.2 Rearing of silkworms

The AMT was weighed and dissolved in DI water to obtain Mo dosages of 0.05, 0.1, 0.5, 1, 5, and 10 g/L in the prepared solutions. Fresh mulberry leaves were rinsed with DI water and air-dried at room temperature until the surface was free of residual water. Then, 200 g of mulberry leaves were weighed, and 30 ml of AMT solution containing various dosages of Mo was sprayed evenly on each side of the mulberry leaves. Subsequently, the moisture on the mulberry leaves were dried at room temperature, and the leaves were preserved in a refrigerator at 4°C for later use.

Silkworms were reared with mulberry leaves at 25°C from the first to the fourth instar, three times per day. On the first day of the fifth instar, silkworm larvae were divided into seven groups of 50 larvae each, as listed in Supplementary Table S1.

### 2.3 Effects of AMT on the growth of the silkworm larvae and their cocooning

From the first day of the fifth instar, the weight of ten silkworms selected randomly from each group was measured. The appearance of the silkworms on the eighth day of the fifth instar was recorded using a camera (D3400, Nikon, Japan). After harvesting the cocoons, the cocooning rate of each group was calculated. In addition, the length and width of each cocoon were measured using calipers. The cocooning rates and cocoon-shell ratios were determined using the following equations:

$$Cocooning\;rate=\frac{n_{1}}{n_{2}}\times 100\%$$ (1)

$$Cocoon\;shell\;ratio=\frac{m_{1}}{m_{2}}\times 100\%$$ (2)

where *n*_*1*_ is the number of cocoons harvested; *n*_*2*_ is 45, the number of silkworms reared from the five silkworms selected for dissection; *m*_*1*_ is the mass of the cocoon shell (g); and *m*_*2*_ is the mass of the whole cocoon (g).

### 2.4 Effects of AMT on the morphology and histology of silk glands

On the eighth day of the fifth instar, five silkworms were randomly selected from each group immediately before cocooning. The silk glands were dissected and rinsed three times with physiological saline before imaging with a camera.

Subsequently, the posterior silk glands were removed and immersed in formaldehyde solution (4%, v/v) for fixation. The posterior silk glands were then embedded in paraffin and sliced (5 μm thick) using a microtome (RM2235; Leica Microsystems Co., Ltd., Germany). For histopathology, the posterior silk glands were stained with H&E and observed under an optical microscope (DM3000, Leica Microsystems Co., Ltd., Germany).

### 2.5 Preparation and characterization of degummed silk

Silk cocoons were degummed by boiling a solution of Na_2_CO_3_, according to a previously published method (Liang et al., 2020). The resultant degummed silk fibers were observed using scanning electron microscopy (SEM; TM4000Plus, HITACHI, Japan) and transmission electron microscopy (TEM; Talos F200X, Thermo Scientific, United States). Prior to the SEM observation, the specimens were sputter-coated with gold to achieve high conductivity. For TEM observation, the degummed silk fibers were cut into fine pieces and sonicated in ethanol for 10 min. A drop of the dispersion was subsequently placed on a carbon-coated copper grid and dried in an oven at 60°C.

Samples were prepared for FTIR analysis following the KBr pellet method (Liang et al., 2020). Each sample was scanned 32 times, from 400 cm^−1^ to 4,000 cm^−1^, with a resolution of 4 cm^−1^. Quantitative analysis of the protein secondary structure was performed for the amide I band from 1,600 cm^−1^ to 1,720 cm^−1^ using a Fourier-transform infrared (FTIR) spectrometer (Nicolet iN10, Thermo Scientific, United States). The baseline of the amide I band was first corrected, and the peak was fitted with a Gaussian function using Origin 9.1 Pro software (Origin Lab, United States).

### 2.6 Molybdenum content determination

Both the degummed silk fibers and cocoons were cut into small pieces, rinsed with DI water three times, and then dried in an oven at 60°C. Based on a previously reported method (Liang et al., 2021), the molybdenum (Mo) contents of both the degummed silk fiber and cocoon were analyzed using inductively coupled plasma optical-emission spectrometry (ICP-OES; Agilent 730, Agilent Technologies, United States).

### 2.7 Silk reeling and mechanical property evaluation

Six cocoons from each group were selected randomly, and each cocoon was placed in boiling water, untangled, and processed using a cocoon-reeling apparatus (YG731, Changzhou First Textile Equipment Co., Ltd., China). For each cocoon, 100 silk strands were reeled and combined to form a single thread. One meter of initially reeled silk thread was cut into three segments. For each segment, the widths of the front, middle, and rear parts were measured at six different positions using optical microscopy. The average width of each thread was calculated from 18 measurements.

Prior to testing, the reeled silk threads were maintained in an artificial climate chamber (HQH-250, Shanghai Yuejin Medical Instrument, China) at 20°C and 65% relative humidity for 24 h. The stress–strain curve of each silk thread was then measured using an electronic tensile-strength measuring machine (YG020, Changzhou First Textile Equipment Co., Ltd., China) according to the China National Standard GB/T 1798-2008 (Standardization Administration of China, 2009). The fracture strength and elongation at break were calculated using Eqs 3, 4. The area under the stress-strain curve was integrated using Origin software to obtain the toughness modulus of each silk thread.

The fracture strength was calculated using the following equation:

$$\sigma =\frac{F}{C}\times 100\%$$ (3)

where *σ* is the fracture strength (MPa), *F* is the force applied parallel to its length (N), and *C* is the cross-sectional area of the silk fiber (cm^2^).

The elongation at break was calculated using the following equation:

$$\varepsilon =\frac{L-L_{0}}{L_{0}}\times 100\%$$ (4)

where *ε* is the elongation at break (%), *L*_*0*_ is the original length of the silk fibers (cm), and *L* is the length of the silk fiber at break (cm).

### 2.8 Preparation and characterization of carbonized silk fibers

The carbonized silk fibers (CSF) were prepared based on a previously reported heating protocol (Liang et al., 2020). The micromorphology and structure of the CSF were observed using SEM and TEM following the procedure described in Section 2.5. It should be noted that sputtering the CSF is unnecessary owing to their inherent electroconductivity.

Subsequently, the Raman spectra of each group were obtained using a Raman spectrometer (inVia, Renishaw, United Kingdom). The analysis was performed at an excitation wavelength of 532 nm, scanning range of 100–3,500 cm^−1^, and spectral resolution of 2 cm^−1^. All data were processed using Origin software. Following a previously reported method (Sadezky et al., 2005; Khatibi et al., 2019), a mixed Gaussian–Lorentzian peak morphology was used to deconvolve the experimental Raman spectrum into a Gaussian-shaped D3 band (1,500 cm^−1^), Lorentzian-shaped D (1,350 cm^−1^), D2 (1,620 cm^−1^), D4 (1,200 cm^−1^), and G bands. As the G band spans from 1,585 cm^−1^ to 1,600 cm^−1^, 1,585 cm^−1^ was chosen to best fit the Lorentzian-shaped band. The relative intensity (peak area) ratio of the D and G bands was calculated.

### 2.9 Electrical conductivity test

The CSF was cut into fine pieces for electrical conductivity measurements following the method reported by Liang et al. (Liang et al., 2020). A Keithley source meter (2,400, Tektronix, United States) and homemade apparatus (Supplementary Figure S1) were used for the test. The electrical conductivity was calculated using the following equation:

$$\rho =\frac{R}{L}\times S$$ (5)

$$\sigma =\frac{1}{\rho }$$ (6)

where *ρ* is the resistivity (Ω·m), *R* is the measured resistance (Ω), *S* is the cross-sectional area of the container containing the packed sample (m^2^), *L* is the length of the sample packed within the container (m), and *σ* is the electrical conductivity (S/m).

### 2.10 Electrochemical measurements

The preparation of a nickel-foam-supported carbonized silk anode is described in the third section of the Supplementary Materials. Cyclic voltammetry (CV), galvanostatic charge–discharge (GCD), and electrochemical impedance spectroscopy (EIS) measurements were performed using an electrochemical workstation (CS350H, Wuhan Corrtest Instruments, China). All these analyses were performed in a 6 M KOH aqueous electrolyte using a three-electrode system composed of a nickel-foam composite as the working electrode, Hg/HgO as the reference electrode, and Pt wire as the counter electrode. CV measurements were performed in a potential window from −1 V to −0.2 V at scan rates of 5, 10, 20, 40, 60, and 80 mV/s. The GCD curves were recorded within a potential window from −1 V to −0.2 V at various current densities, including 0.2, 0.4, 0.6, 0.8, 1, and 2 A/g. The specific capacitance of each sample was calculated using the following equation:

$$C_{m}=\frac{I\times ∆t}{M\times ∆V}$$ (7)

where *C*_*m*_ (F/g) is the mass specific capacitance, *I* (A) is the discharge current, Δ*t* (min) is the discharge time, *M* (g) is the mass of the CSF loaded on the nickel foam, and Δ*V* (V) is the potential window.

## 3. Results and discussion

### 3.1 Investigation of AMT-fed silkworm growth and cocooning

**FIGURE 1:**
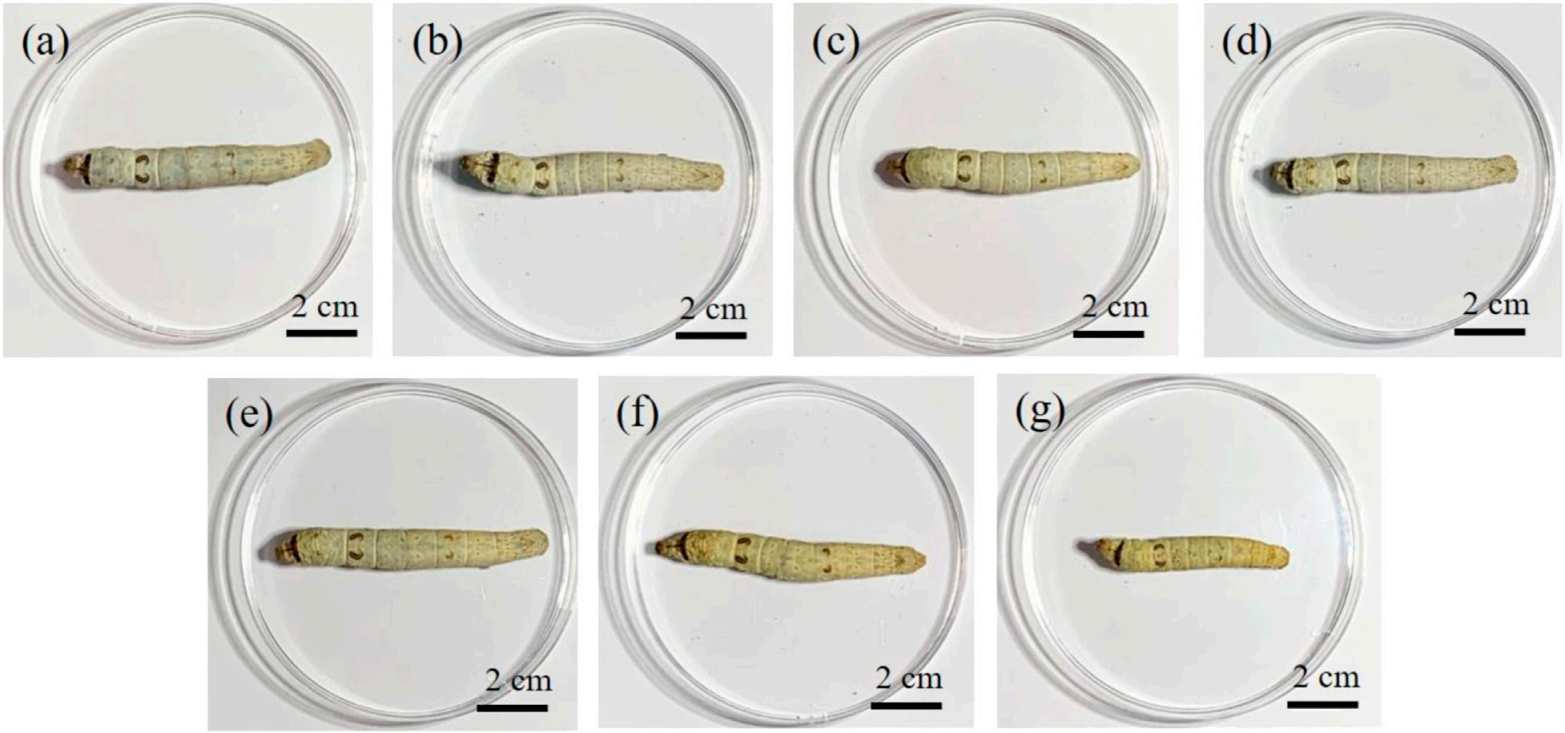
Effect of AMT feeding on the appearance of the silkworm larvae: **(A)** control group, **(B)** Mo-0.05 g/L group, **(C)** Mo-0.1 g/L group, **(D)** Mo-0.5 g/L group, **(E)** Mo-1 g/L group, **(F)** Mo-5 g/L group, and **(G)** Mo-10 g/L group.

The effect of AMT on the growth of silkworm larvae was investigated. As shown in Figure 1, the body lengths of the silkworms from the Mo-5 g/L and Mo-10 g/L groups were much shorter than those of the other groups. Combining the experimental data for silkworm growth (Supplementary Figure S3), it can be concluded that the AMT-solution-sprayed mulberry leaves fed to both Mo-5 g/L and Mo-10 g/L groups exerted a strong toxic effect on the silkworm.

It was found that only a few silkworms in the Mo-5 g/L and Mo-10 g/L groups could survive through cocooning and began to pupate, resulting in a low cocooning rate (Supplementary Figure S4). In addition, it is noteworthy that the cocoons spun by the surviving silkworm in both the Mo-5 g/L and Mo-10 g/L groups were unreelable owing to their very thin cocoon shells (Supplementary Figure S5), thereby exhibiting a poor economic value. Therefore, the Mo-5 g/L and Mo-10 g/L groups were not included in the subsequent experiments. Fortunately, the silkworm in the control, Mo-0.05 g/L, Mo-0.1 g/L, Mo-0.5 g/L, and Mo-1 g/L groups displayed acceptable cocooning rates (Supplementary Figure S4).

**FIGURE 2:**
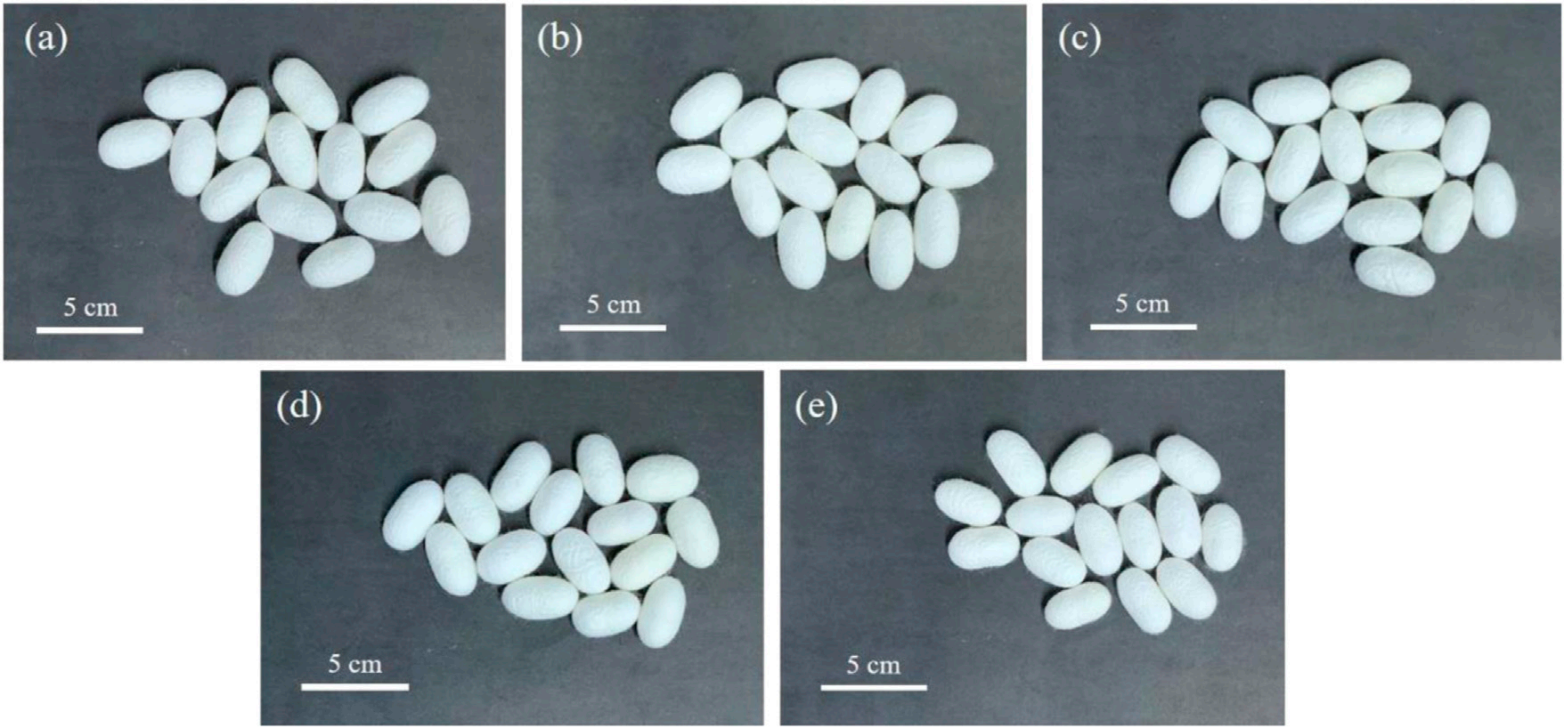
Effect of AMT feeding on the appearance of silkworm cocoons: **(A)** control group, **(B)** Mo-0.05 g/L group, **(C)** Mo-0.1 g/L group, **(D)** Mo-0.5 g/L group, and **(E)** Mo-1 g/L group.

**FIGURE 3:**
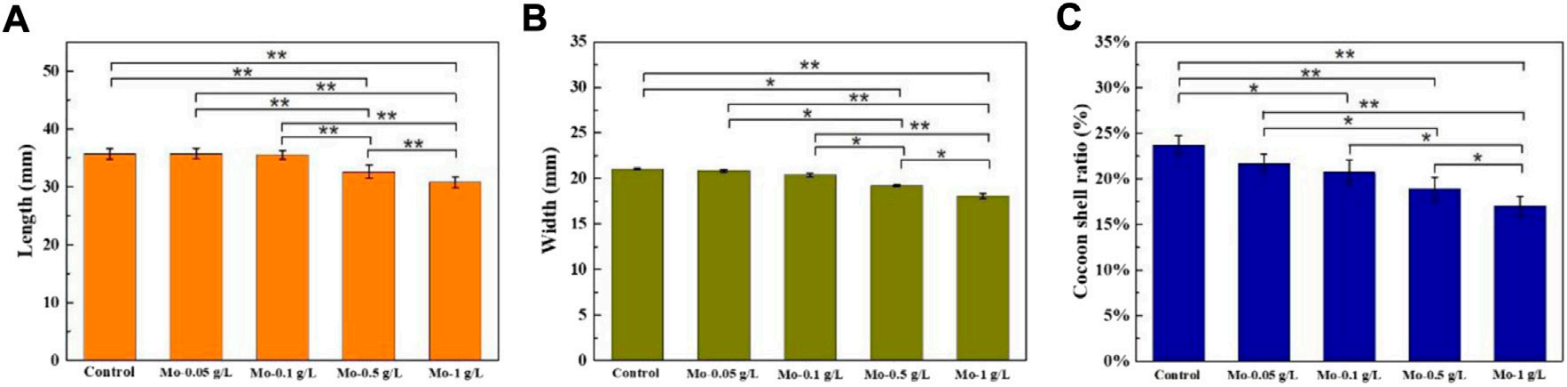
Effect of AMT feeding on the cocoon **(A)** length, **(B)** width, and **(C)** shell ratios. (n = 5, * *p* < 0.05, ** *p* < 0.01).

A further investigation of the cocoons of the silkworms from the control, Mo-0.05 g/L, Mo-0.1 g/L, Mo-0.5 g/L, and Mo-1 g/L groups, as shown in Figure 2, revealed that the width and length of the cocoon, as well as the cocoon shell ratio, declined with an increase in AMT dosage fed to the silkworm (Figure 3). This finding suggests that AMT had a negative effect on silkworm cocooning.

### 3.2 Morphology and histology of the silk gland

**FIGURE 4:**
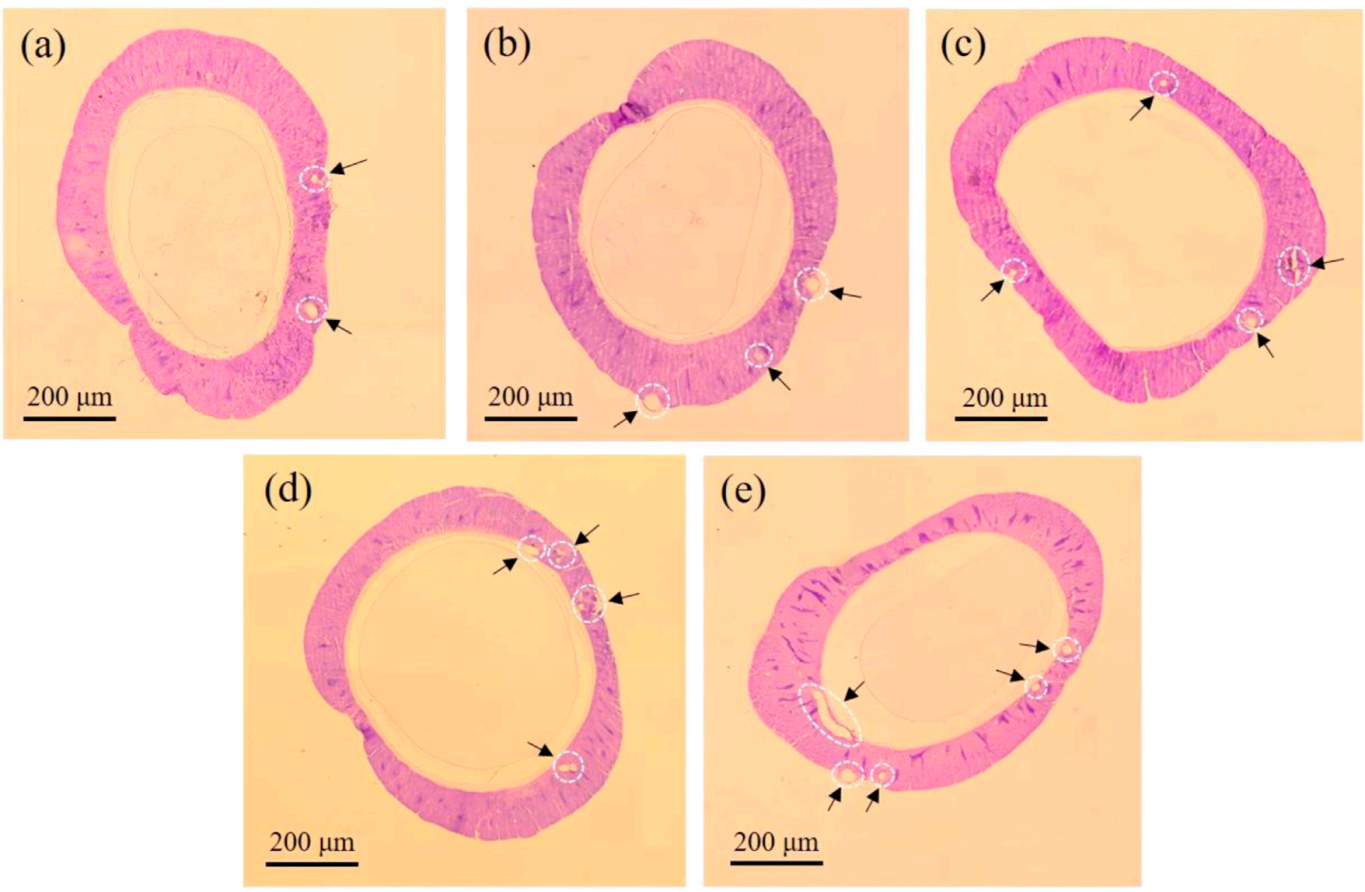
Histophysiological image of posterior silk gland from **(A)** control group, **(B)** Mo-0.05 g/L group, **(C)** Mo-0.1 g/L group, **(D)** Mo-0.5 g/L group, and **(E)** Mo-1 g/L group. The dark arrows point to the areas of vacuolation.

The posterior silk gland (PSG) is the only organ that synthesizes and secretes silk fibroin (Hou et al., 2007). Here, H&E-stained PSG histopathological images were used to understand the impact of AMT on PSG development. Silkworm silk-gland tissue includes the outer epithelial membrane, gland cells, and inner epithelial membrane (Akai, 1983). Among these, gland cells are involved in the secretion of silk proteins. The number of gland cells is positively correlated with the silk production quantity (Wu et al., 1961). Therefore, the sparseness of silk gland cells reflects the degree of silk gland injury (Cheng et al., 2018). As shown in Figure 4, the gland cell population decreased with increasing AMT feeding dosage, and the H&E-stained section of the Mo-1 g/L group demonstrated the most severe vacuolation. Such damage may be responsible for the decreased cocoon shell ratio and cocoon size as the AMT feeding amount increased.

In addition to PSG, adverse effects caused by feeding silkworms high dosages of AMT were apparent in the anterior and middle silk glands, as shown in Supplementary Figure S6. The results indicate that feeding silkworms with a high dosage of AMT suppressed silk gland development.

### 3.3 Mo content determination

Degummed silk is commonly used in the textiles industry. ICP-OES was employed to determine the elemental Mo content remaining in the degummed silk. As shown in Supplementary Table S2, compared with the Mo content in the control group, the Mo content in the degummed silk continued to increase as the AMT feeding dose increased. The Mo content in the cocoons of each group exhibited a similar trend (Supplementary Table S3), indicating that Mo was distributed in both silk sericin (SS) and silk fibroin (SF). These results show that feeding silkworms AMT can effectively enrich the Mo content of silk.

### 3.4 Microstructural study of degummed silk

**FIGURE 5:**
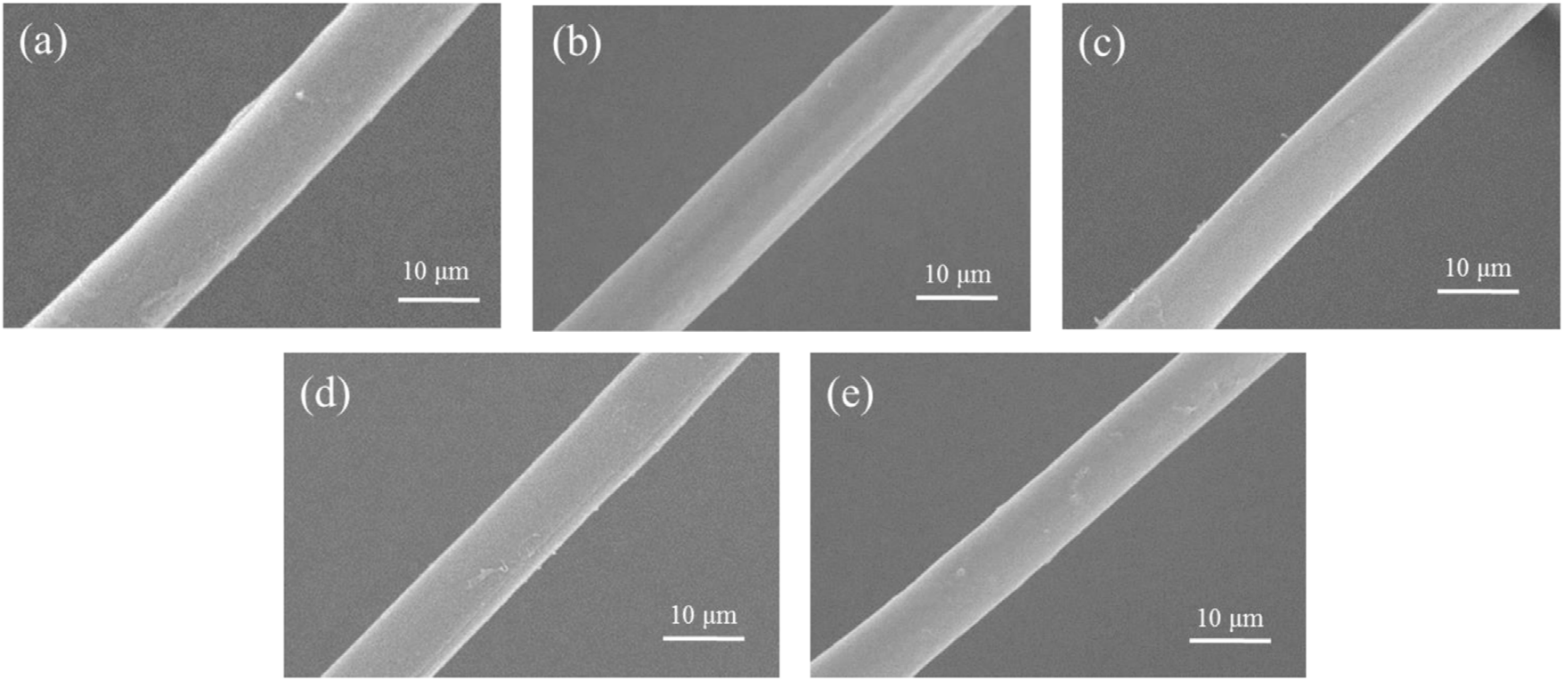
Representative SEM images of the degummed silk fiber from the **(A)** control group, **(B)** Mo-0.05 g/L group, **(C)** Mo-0.1 g/L group, **(D)** Mo-0.5 g/L group, and **(E)** Mo-1 g/L group.

Raw silk consists of two components, SF and SS, where the former endows silk with good mechanical properties and stability (Kim et al., 2005). Figure 5 presents a SEM image showing the micromorphology of degummed silk. It is clear that the morphologies of the degummed silk from each group are similar, but their widths vary. The average width of the degummed silk decreased with increasing AMT dosage (Supplementary Figure S7). We assume this is due to the reduced silk production caused by vacuolation of the silk gland, as discussed in Section 3.2.

### 3.5 Transmission electron microscopy investigation

**FIGURE 6:**
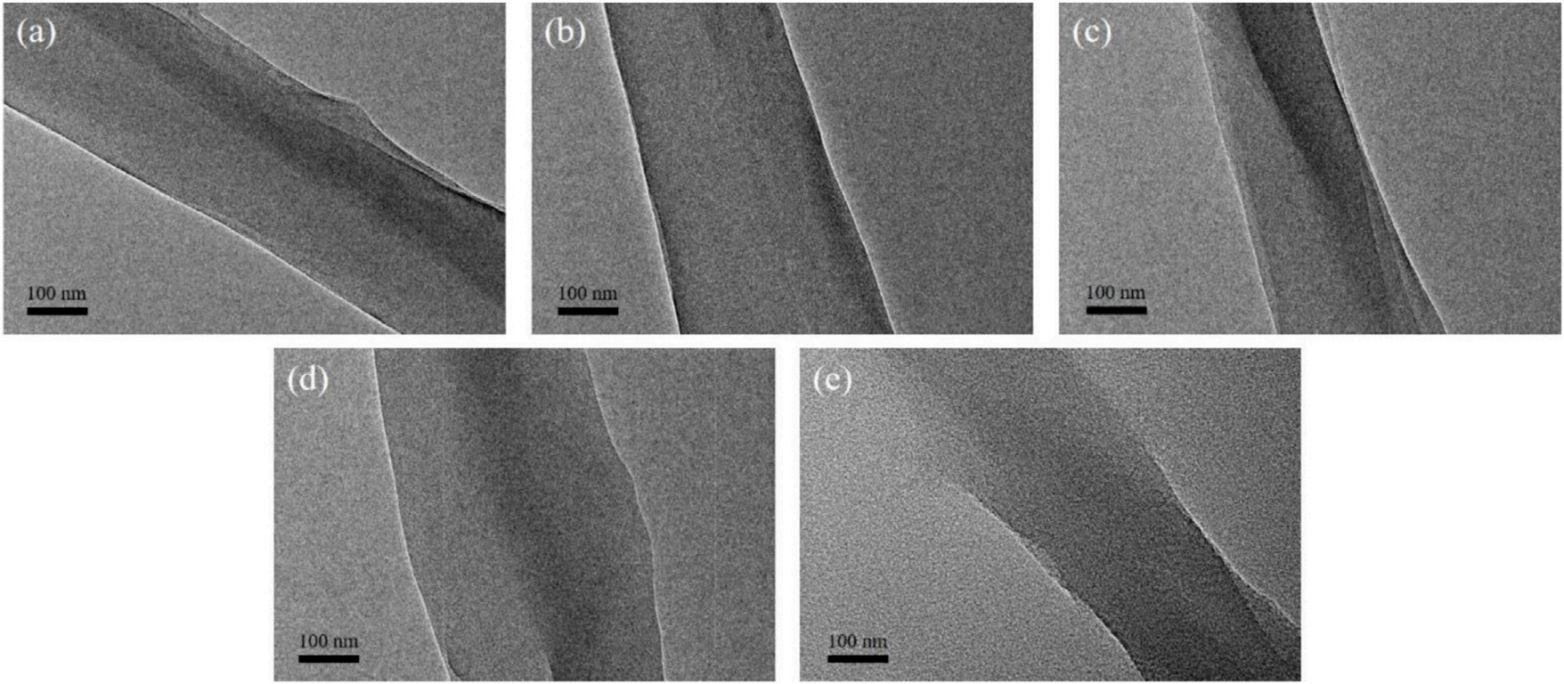
Representative TEM images of the degummed silk fiber from the **(A)** control group, **(B)** Mo-0.05 g/L group, **(C)** Mo-0.1 g/L group, **(D)** Mo-0.5 g/L group, and **(E)** Mo-1 g/L group.

Transmission electron microscopy was used to evaluate the interior structure of degummed silk. As shown in Figure 6, neither particles nor crystal structures were observed within the degummed silk of any group. Whereas EDS analysis confirmed the uniform distribution of Mo along the degummed silk fiber (Supplementary Figure S8, S9). Therefore, we believe that AMT fed to the silkworm entered silk as a precursor.

### 3.6 FTIR spectroscopy characterization of degummed silk

**FIGURE 7:**
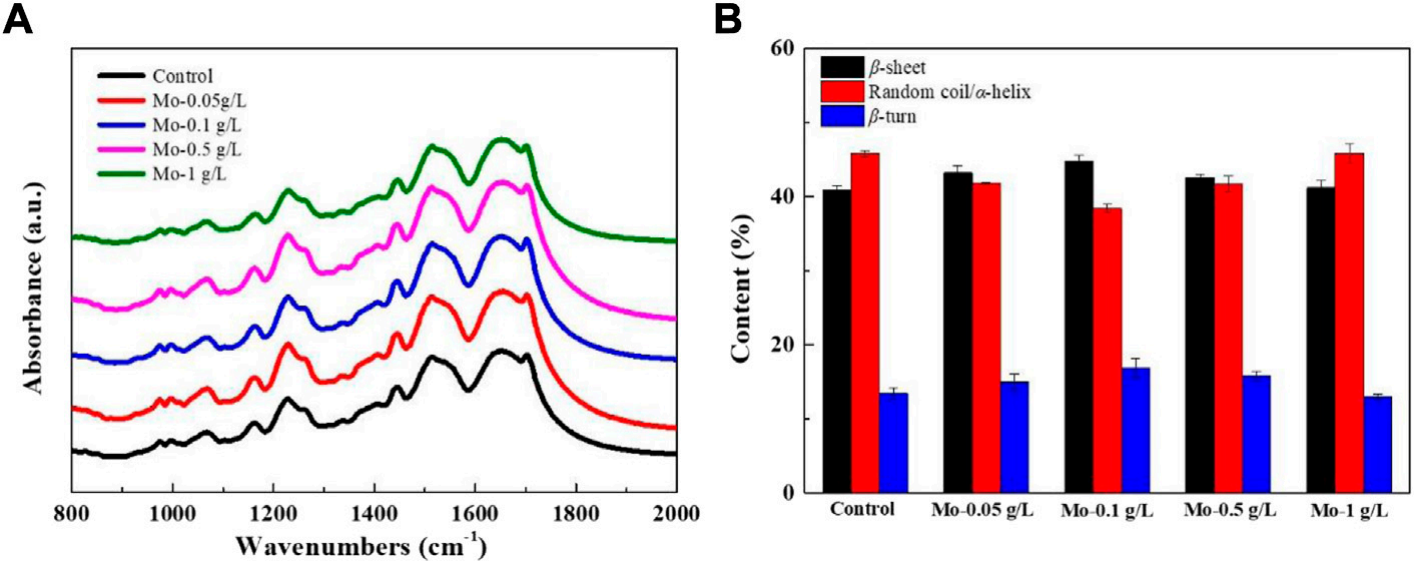
Effect of AMT feeding on the secondary structures of silk fibroin. **(A)** FTIR spectra of silk fibroin from each group, and **(B)** the percentage contents of secondary structures in the amide I band of silk fibroin from each group.

The mechanical behavior of silk fiber is expected to be highly dependent on its secondary structure (Wang et al., 2017). Here, FTIR spectroscopy was used to determine the distribution of the secondary structure in the degummed silk to explore the effect of feeding AMT to each group and to provide detailed information on the mechanical properties of the silk. As shown in Figure 7A, each group shares a similar protein molecular structure profile containing three different amide groups: amide I (1,600–1720 cm^−1^), amide II (1,500–1,600 cm^−1^), and amide III (1,200–1,350 cm^−1^) (Cheng et al., 2018; Chen et al., 2019). Deconvolution of the amide I band of each group (Supplementary Figure S10) revealed variations in the percentages of *β*-sheets, random coils/*α*-helixes, and *β*-turns (Figure 7B). The composition of the secondary structure (Supplementary Table S4) revealed that the percentages of both *β*-sheets and *β*-turns increased once AMT was fed to the silkworm and was maximal when the Mo feeding dosage reached 0.1 g/L. This value then decreased when the Mo feeding dosage was greater than 0.1 g/L. By contrast, the percentages of random coils/*α*-helixes exhibited the opposite trend, declining initially, subsequently increasing, and then reaching its minimum value when the Mo feeding dosage was 0.1 g/L.

Metal ions can coordinate with silk fibroin, promoting its conformational transition (Xu and Zhang, 2008; Liu et al., 2019). We believe that a low concentration of molybdate can induce the formation of *β*-sheets and *β*-turns through coordination and chelation with SF, thereby helping to align and orient SF molecular chains. In addition, the formation of intermolecular hydrogen bonds among the SF molecules facilitates the transformation of the SF secondary structure (Viles et al., 1999). Thus, when the Mo feeding dosage ranged from 0.05 g/L to 0.1 g/L, we observed a rise in the percentage distributions of both *β*-sheets and *β*-turns, while the percentage of random coils/*α*-helixes declined. It is reasonable to infer that the repulsive force arising from the excess molybdate coordinated with SF would inhibit the interactions among SF molecular chains, thus inhibiting its conformational transition. Such an inference can explain the decrease in the percentage distributions of both *β*-sheets and *β*-turns and increase in the percentage of random coils/*α*-helixes when the AMT feeding dosage ranged from 0.5 g/L to 1 g/L. A schematic representation of this process is shown in Supplementary Figure S11.

### 3.7 Determination of silk-thread mechanical properties

Before the mechanical tests were carried out, the silk threads were observed using an optical microscope and their widths were measured, as presented in Supplementary Figure S12. According to the analysis (Supplementary Figure S13), the width of the silk thread from each group decreased continuously as the AMT feeding dosage increased. This trend is consistent with the variation of the degummed silk width with AMT feeding dosage (Supplementary Figure S7).

**FIGURE 8:**
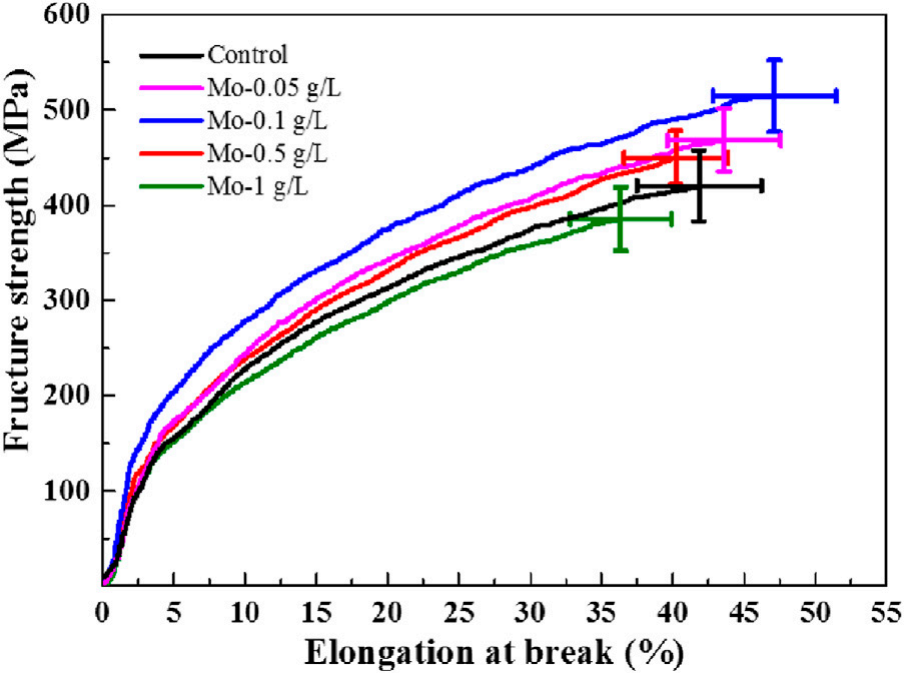
Strain–stress curves of the obtained silk threads.

Subsequently, the mechanical properties of silk threads from each group were evaluated by performing tensile tests, with results from all testing summarized in Supplementary Table S5–S9. The stress–strain curves are shown in Figure 8. It can be observed that both the fracture strength and elongation at break initially increased as the AMT feeding dosage increased, and both reached a maximum when the Mo feeding dosage reached 0.1 g/L. Subsequently, both the fracture strength and elongation at break decreased when the Mo feeding dosage was greater than 0.1 g/L.

The effect of AMT on the mechanical properties of silk can be attributed to the secondary structure of silk fibroin. As described in Section 3.6, feeding silkworms ≤0.1 g/L of Mo can promote the formation of *β*-sheets and *β*-turns (Xu and Zhang, 2008). It is known that the *β*-sheet structure imparts stiffness and toughness to silk (Porter and Volrath., 2010; Mandal et al., 2012); therefore, the enhanced percentage distribution of the *β*-sheet structure would lead to a higher fracture strength of the silk thread. On the other hand, folding the *β*-sheet structure back onto itself is a function of the *β*-turn structure, which can facilitate the formation of crystalline *β*-sheet stacks stabilized by hydrogen bonds and Van der Waals interactions between sheets (Nelson and Cox., 2005). Such weak interactions are responsible for the flexibility and softness of silk, thereby increasing its elongation at break. When the Mo feeding dosage exceeded 0.1 g/L, the repulsion from the protein-bound molybdate inhibited the secondary structural transition of silk from random coil/*α*-helix to *β*-sheet and *β*-turn structures, as well as the interactions among all peptides, resulting in a decline in fracture strength and elongation at break.

With the molybdate concentration is further increased, electrostatic repulsion among molybdate–silk fibroin complexes can be expected, hindering the aggregation of molybdate-bound silk fibroin and ultimately inhibiting the transformations of *α*-helixes/random coils into *β*-sheet structures. The decrease in *β*-sheet content caused the silk fibers to break easily. Moreover, electrostatic repulsion may affect the supramolecular interactions of the *α*-helix/random coil itself, such as hydrogen bonds and Van der Waals forces, unfolding the amorphous chains and ultimately affecting the flexibility of the silk fibers (Pérez-Rigueiro et al., 2000).

The modulus of toughness can be calculated from the integral area under the stress–strain curve and represents the total energy absorbed by the silk thread before it fractures (Vollrath and Porter 2006). As shown in Table 1, the modulus of toughness for the silk thread from each group followed a similar trend to those of both fracture strength and elongation at break, reaching its maximum value when the Mo feeding dosage was 0.1 g/L.

**TABLE 1:** Mechanical properties of the obtained silk threads.

	Properties		
Sample	Fracture strength [MPa]	Elongation at break [%]	Modulus of toughness [GJ/m3]
Control	418.88 ± 37.04	42.06 ± 4.36	11.80 ± 1.54
Mo-0.05 g/L	468.29 ± 33.32	44.03 ± 3.97	14.20 ± 2.44
Mo-0.1 g/L	508.46 ± 37.66	46.22 ± 4.32	16.02 ± 1.83
Mo-0.5 g/L	459.30 ± 28.16	41.26 ± 3.63	12.57 ± 0.94
Mo-1 g/L	399.69 ± 33.48	37.22 ± 3.55	9.95 ± 1.18

### 3.8 SEM and TEM analyses of carbonized silk fibers

**FIGURE 9:**
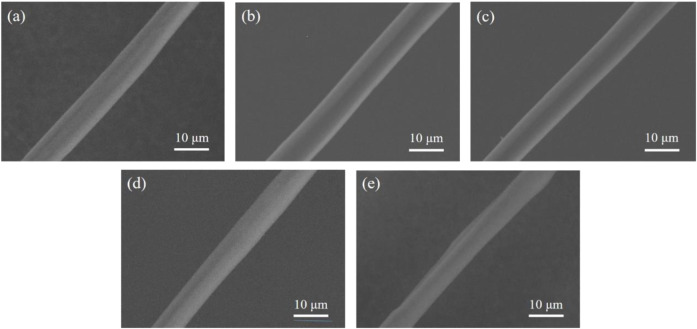
Representative SEM images of CSF derived from the **(A)** control group, **(B)** Mo-0.05 g/L group, **(C)** Mo-0.1 g/L group, **(D)** Mo-0.5 g/L group, and **(E)** Mo-1 g/L group.

SEM was used to observe the morphology of the CSF derived from each group. As shown in Figure 9, the degummed silk remained fibrous after carbonization. In addition, the width of the CSF is smaller than that of the degummed silk, which can be attributed to the pyrolysis process (Fisher et al., 2002). It should be noted that the SEM images were acquired without coating the sample with gold owing to the good conductivity of CSF.

**FIGURE 10:**
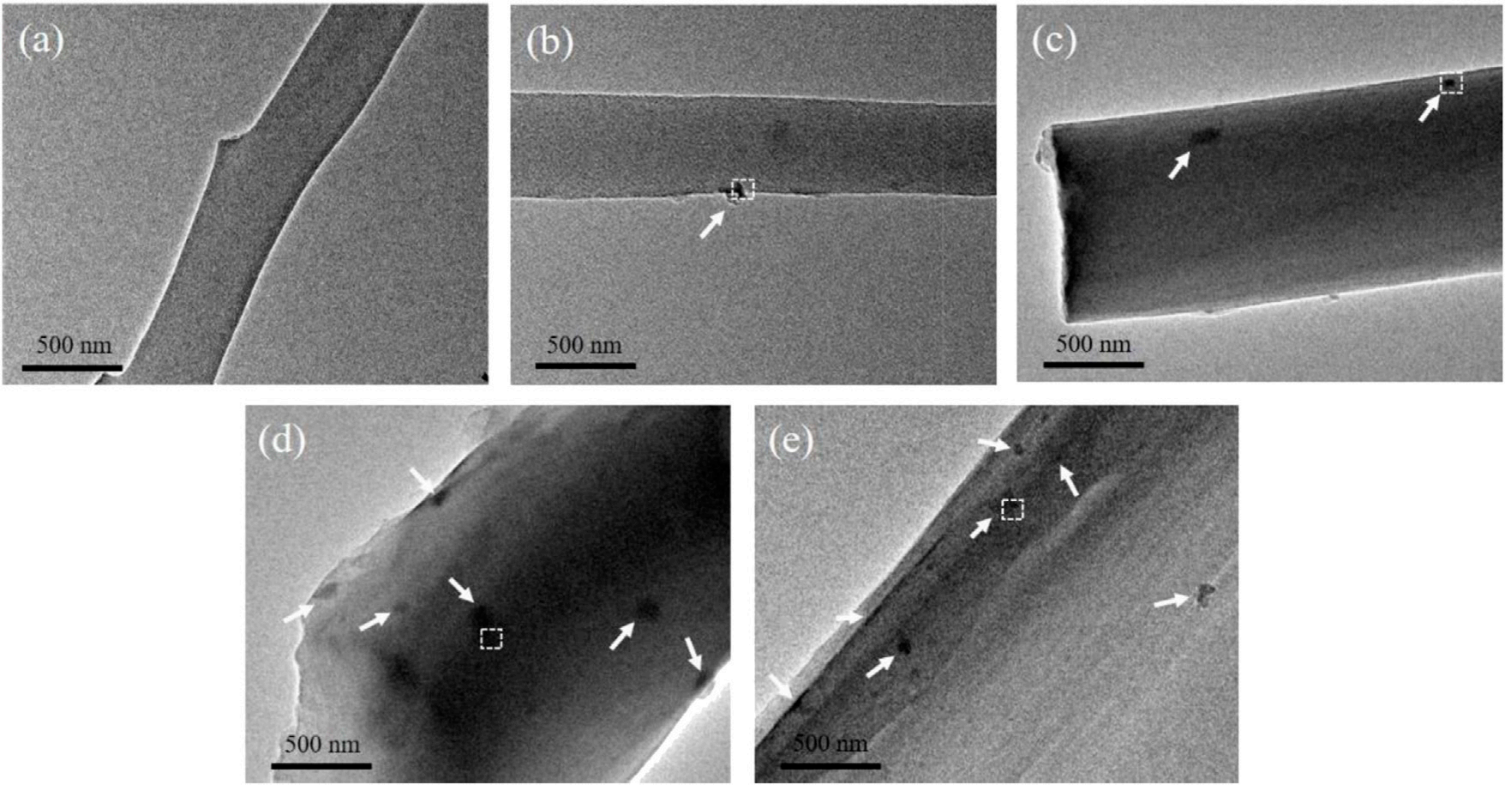
Representative TEM images of CSF derived from **(A)** control group, **(B)** Mo-0.05 g/L group, **(C)** Mo-0.1 g/L group, **(D)** Mo-0.5 g/L group, and **(E)** Mo-1 g/L group. The white arrows point to nanoparticles.

TEM was used to characterize the structure and composition of CSF. As shown in Figure 10, nanoscale opacities or “dark spots” are visible within the CSF of each AMT feeding group. In addition, the number of dark spots associated with each group gradually increased with an increase in AMT feeding dosage. By contrast, no dark spots were observed within the CSF of the control group.

**FIGURE 11:**
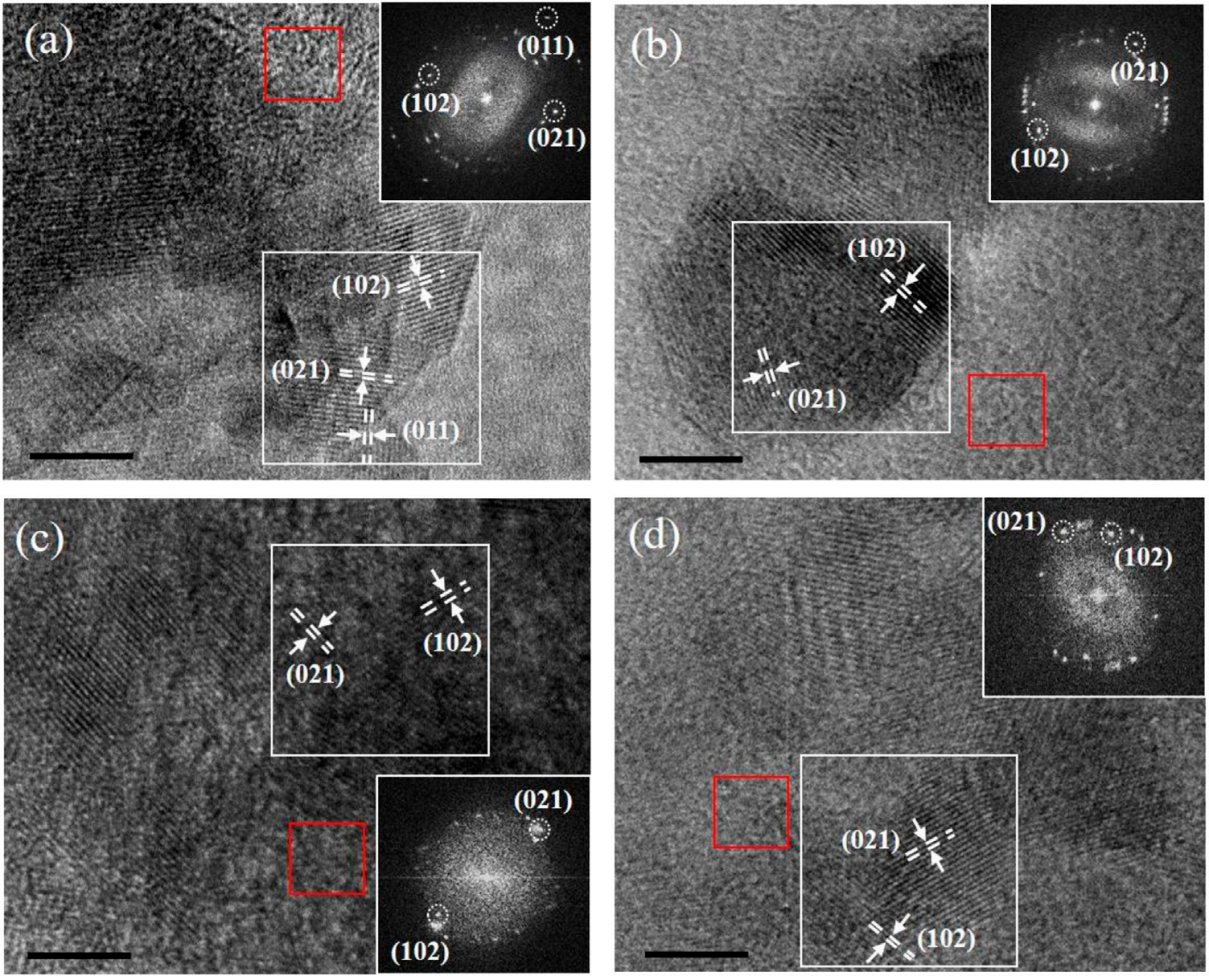
Representative HRTEM images of nanoparticles from **(A)** Mo-0.05 g/L group, **(B)** Mo-0.1 g/L group, **(C)** Mo-0.5 g/L group, and **(D)** Mo-1 g/L group. The insets show the corresponding FFT images of the areas framed by the white boxes (scale bar = 5 nm).

High-resolution TEM (HRTEM) was used to elucidate the crystallographic orientation of the inclusions, which appear as dark spots in the images shown in Figure 10. Figure 11 displays the magnified HRTEM images of the particles delineated by the white dotted boxes in Figure 10. The lattice-resolved HRTEM images of the particles embedded in the CSF display interplanar spacings of 0.152, 0.231, and 0.275 nm, corresponding to the (011), (021), and (102) planes, respectively, of MoO_2_ (JCPDS 73-1249). Amorphous carbon derived from SF can also be observed, as indicated by the solid red boxes in each image. Furthermore, the insets show the fast Fourier transforms (FTT) of the areas within the white solid boxes in each image; the (011), (021), and (102) lattice planes of MoO_2_ can be identified. Additionally, the EDS analysis confirmed that the dark spots embedded in the CSF contained Mo (Supplementary Figure S14).

Since silk fiber consists of a bundle of nanofibrils with a geometric width ranging from 90 to 170 nm (Putthanarat et al., 2000), we believe that this architecture limits the growth and aggregation of MoO_2_ particles, resulting in the uniform dispersion of MoO_2_ NPs in the silk, as shown in Figures 10, 11.

### 3.9 Raman characterization of carbonized silk fibers

**FIGURE 12:**
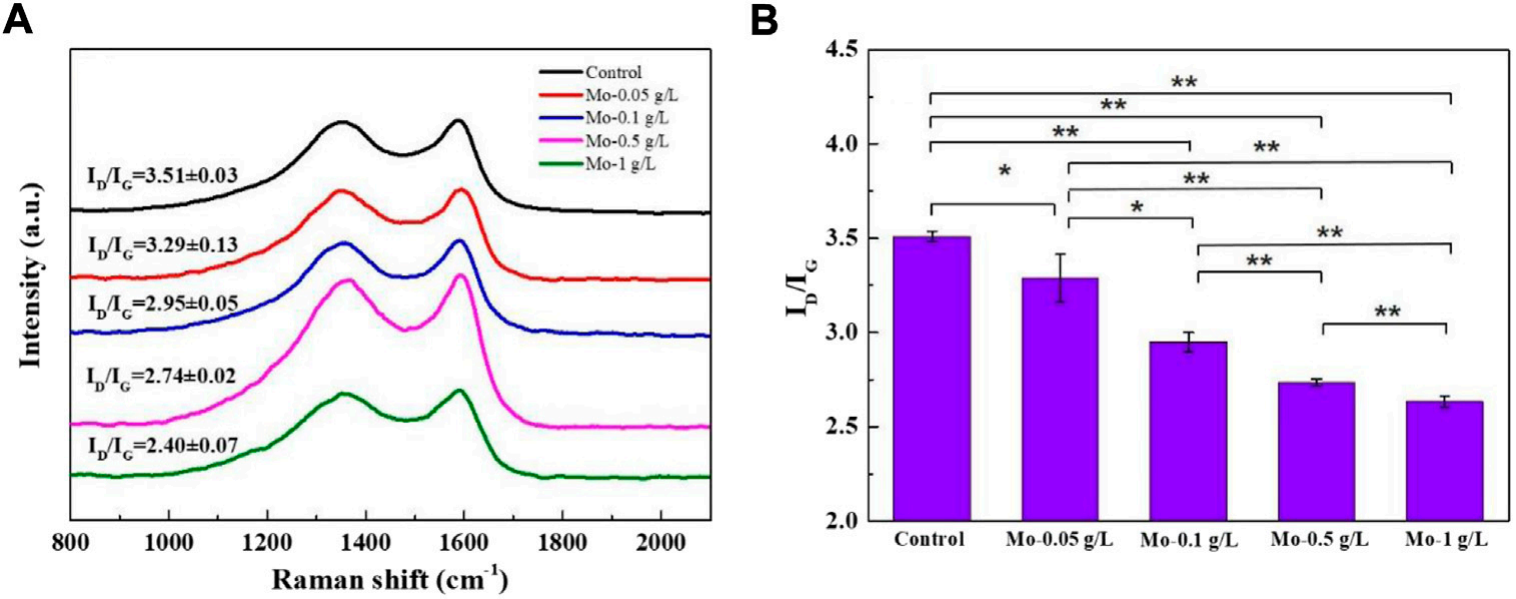
Effect of MoO_2_ NP incorporation on the graphitization degree of CSFs: **(A)** Raman spectra and **(B)** I_D_/I_G_ value of CSFs from each group (n = 3, * *p* < 0.05, ** *p* < 0.01).

To investigate the effect of MoO_2_ NPs generated *via* pyrolysis on the graphitization degree of CSFs, the Raman spectra of the CSFs from each group were deconvoluted (Supplementary Figure S15) and analyzed. As shown in Figure 12A, the Raman spectra of each group display two characteristic peaks at 1,350 cm^−1^ (D band) and 1,585 cm^−1^ (G band). The D band represents the defects and disorder in the carbon lattice, while the G band represents the in-plane stretching vibration of *sp* ^*2*^-hybridized carbon atoms in the graphite layers (Yang et al., 2020). The graphitization degree can be determined by the relative intensity ratio of the D and G bands, I_D_/I_G_, where a lower I_D_/I_G_ value indicates a higher graphitization degree (Dong et al., 2014).

As shown in Figure 12B, the I_D_/I_G_ value of the CSFs gradually decreased as the AMT feeding dosage increased, and the CSF derived from the Mo-1 g/L group possessed the highest graphitization degree among all the samples. Based on the fact that MoO_2_ NPs can induce the phase transformation of amorphous carbon into graphite (Liu et al., 2014), we believe that the growing number of MoO_2_ NPs generated during carbonization progressively improved the graphitization degree of the CSFs.

The conductivity of the CSF derived from each group generally supports the variation in graphitization degree indicated by the Raman spectra, as it has been reported that carbonaceous materials with higher graphitization degrees tend to have higher electrical conductivities (Hou Y. et al., 2021). It can be seen from Supplementary Table S10 that the conductivity of the CSF started to rise when the Mo feeding dosage was 0.1 g/L, reaching its maximum at a dosage of 1 g/L. The difference in conductivities of CSF derived from the control, Mo-0.05 g/L, and Mo-0.1 g/L groups were insignificant, which might be attributed to the relatively small difference in graphitization degree among those samples.

### 3.10 Electrochemical test

**FIGURE 13:**
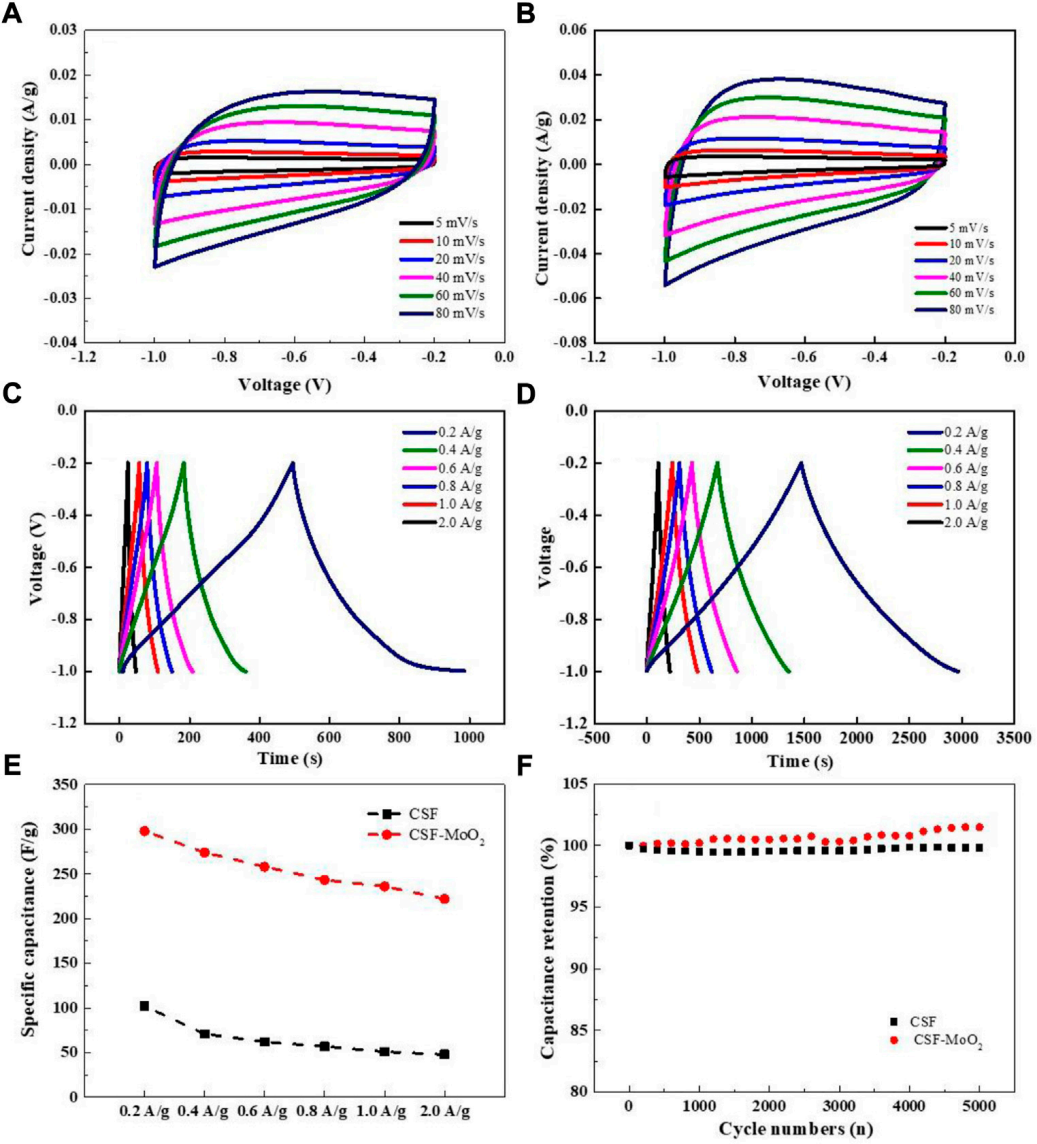
Capacitance characterization of electrode prepared from the carbonized silk. CV curves of **(A)** CSF and **(B)** CSF-MoO_2_ electrodes at different scan rates. GCD curves generated at current densities of 0.2–2 A/g for **(C)** CSF and **(D)** CSF-MoO_2_ electrodes. **(E)** Specific capacitances of CSF and CSF-MoO_2_ electrodes at different current densities. **(F)** Cycling stability of CSF and CSF-MoO_2_ electrodes at a current density of 2 A/g.

The CSFs derived from the control group (CSF) and Mo-1 g/L group (CSF-MoO_2_) were evaluated as electrode materials for supercapacitors. Figures 13A,B show the CV curves of the electrodes prepared from CSF and CSF-MoO_2_. The curves exhibit a quasi-rectangular shape, indicating that the mechanism of charge storage can be attributed to the electric double-layer capacitance (EDLC) and pseudo-capacitance. This pseudo-capacitance behavior is due to the doping of CSF with nitrogen sourced from the SF protein (Sahu et al., 2015; Li et al., 2016). In addition, it can be seen from Figures 13A,B that with an increase in the scan rate, the response current for both electrodes increases owing to fast ion- and electron-migration rates (An et al., 2019). Furthermore, the area enclosed by the CV curves of the CSF-MoO_2_ electrode is greater than that of the CSF electrode at each scan rate, indicating that the CSF-MoO_2_ electrode has a much better specific capacitance than the CSF electrode (Sun et al., 2021).

The galvanostatic charge–discharge (GCD) curves at different current densities from 0.2 A/g to 2.0 A/g were acquired for the CSF and CSF-MoO_2_ electrodes, and the results are presented in Figures 13C,D. The GCD curves of the CSF electrode deviate from the ideal triangular shape (Figure 13C), which confirms the existence of both EDLC and pseudocapacitive behavior (Vijeth et al., 2018; Potphode et al., 2020). In addition, the significant voltage drop at a current density of 2.0 A/g indicates that the CSF electrode has a lower charge and discharge efficiency, with a relatively high internal resistance (Mo et al., 2018).

By contrast, the GCD curves of the CSF-MoO_2_ electrode generated using different applied current densities all show a nearly symmetrical triangular shape (Figure 13D), indicating excellent capacitive performance with a high degree of electrochemical reversibility (Song et al., 2019). Furthermore, the discharge time of the CSF-MoO_2_ electrode was much longer than that of the CSF electrode, revealing a significant improvement in the specific capacitance and energy storage of the electrode (Maher et al., 2021). This enhancement was also supported by the calculated specific capacitance of both electrodes shown in Figure 13E, indicating that the CSF-MoO_2_ electrodes have higher specific capacitances at various current densities compared with those of the CSF electrodes. The CSF-MoO_2_ electrode exhibited a maximum capacitance of 298 F/g at a current density of 0.2 g/A. We believe that the CSF-MoO_2_ electrodes achieve better energy storage capability than the CSF electrodes owing to the generation of MoO_2_ at the nanoscale, which is an essential feature of pseudocapacitive behavior (Hercule et al., 2013; Zhou et al., 2016), as well as the high graphitization degree induced by this nanoscale MoO_2_.

The GCD curves and specific capacitance of the CSF electrodes prepared from groups Mo-0.05 g/L, Mo-0.1 g/L, and Mo-0.5 g/L were measured and demonstrated in Supplementary Figure S16 and Supplementary Table S10, respectively. It can be concluded that the capacitance of the CSF electrodes prepared from various groups increased as the AMT feeding dosage increased.

Maintaining good cycling stability is important for the practical application of supercapacitors (Sharma et al., 2021). Therefore, the cycle retention was determined by performing a GCD test at a current density of 2 A/g for 5,000 cycles. The gradual increase in capacitance of the CSF-MoO_2_ electrode during charging and discharging can be attributed to the penetration of the electrolyte and its contact with MoO_2_ NPs embedded within the CSF. In addition, the high Coulombic efficiency of the CSF-MoO_2_ electrode also demonstrates its long-term cycle-life performance (Supplementary Figure S17).

**FIGURE 14:**
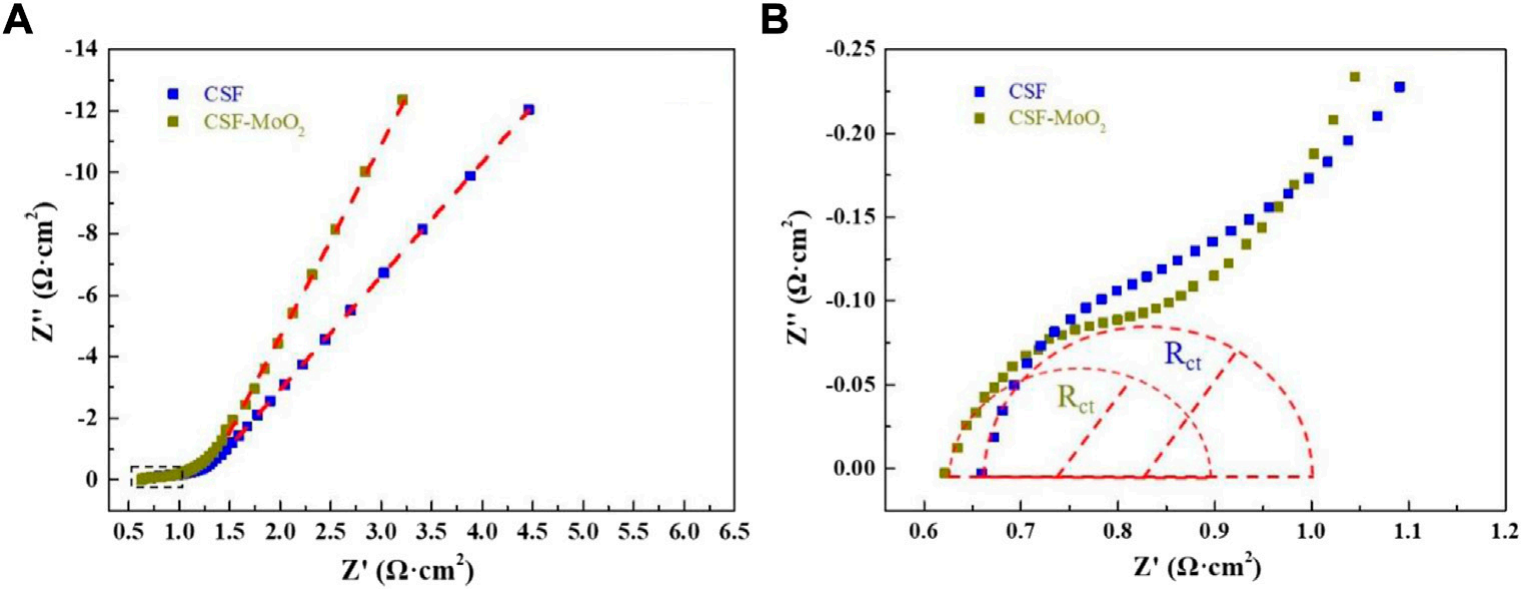
EIS spectra: **(A)** Nyquist plots of the CSF and CSF-MoO_2_ electrodes, and **(B)** magnified image of the area within the dashed box in **(A)**.

The Nyquist plot, representing the impedance characteristic as a function of frequency, was applied to evaluate the kinetics and interfacial resistance of both the CSF electrode and CSF-MoO_2_ electrode (Mo et al., 2018). As shown in Figure 14, the Nyquist plot contains a semicircle in the high-frequency region, followed by a straight line in the low-frequency region. The initial semicircle intercept in the high-frequency region of the real impedance axis provides the internal resistance (R_s_), which includes the resistance of the electrolyte, the intrinsic resistance of the active material, and the electrode/electrolyte interfacial contact resistance. As shown in Figure 14A, the R_s_ of the CSF-MoO_2_ electrode is lower than that of the CSF electrode, indicating that the CSF-MoO_2_ electrode tested in the KOH electrolyte possesses a lower internal resistance.

In addition, the diameter of the semicircle represents the charge-transfer resistance (R_ct_), which can be used to determine the rates of charge and discharge of the supercapacitor and can be considered a very important factor in determining the power densities of the supercapacitor (Mo et al., 2018). Figure 14B reveals that the CSF-MoO_2_ electrode has a smaller diameter than that of the CSF electrode, indicating a smaller R_ct_. Furthermore, the EIS curve of the CSF-MoO_2_ electrode was steeper in the low-frequency region, demonstrating a better capacity for ion transmission in the KOH electrolyte (Sun et al., 2021).

## 4 Conclusion

In summary, AMT-solution-sprayed mulberry leaves were fed to silkworms to introduce the precursor of an electrochemically active substance into silk. It was found that feeding AMT-solution-sprayed mulberry leaves to silkworms had some negative effects on the growth, silk spinning, and cocooning of the silkworms but positively impacted the mechanical strength of the silk fiber when the Mo feeding dosage was less than 0.5 g/L. We confirmed the *in situ* growth of MoO_2_ NPs during the pyrolysis process. Electrochemical experiments revealed that the pseudocapacitor electrodes prepared from silk spun by silkworms in the Mo-1 g/L group had a specific capacitance of 298 F/g, which was higher than that of the control group (102 F/g). This specific capacitance was also higher than the value (245 F/g) obtained by feeding silkworms mulberry leaves sprayed with an aqueous solution of 5 g/L of MoO_2_ NPs, as reported in our previous work (Liang et al., 2020). Remarkably, the cycling stability of the electrode was exceptional, with the specific capacitance remained unchanged after 5000 GCD cycles.

In comparison with feeding nanoparticles to the silkworms, the presented strategy avoids the aggregation of nanoparticles effectively. Especially, the strategy is able to modulate the amount of precursor of the electrochemically active substance that is incorporated within silk fiber, therefore is beneficial for the design of functionalized CSF programmatically. Furthermore, the metallic salt is generally less expensive than its corresponding metallic nanoparticles, and can make a homogenous solution more conveniently, which is promising for the fabrication of carbonized silk with desired property in large-scale.

## Data Availability

The original contributions presented in the study are included in the article/Supplementary Materials, further inquiries can be directed to the corresponding author.
