# Supplementary material for: In situ preparation of molybdenum-dioxide-incorporated carbonized silk fiber and its application in supercapacitors
Source: Front Bioeng Biotechnol. 2022 Nov 17;10:1059399. doi: 10.3389/fbioe.2022.1059399 (PMC9712748; doi:10.3389/fbioe.2022.1059399)
Supplement: Supplementary file 1 [file DataSheet1.docx]

**Supplementary Materials**

**In-situ preparation of molybdenum-dioxide-incorporated carbonized silk fiber and its application in supercapacitors**

**Yansong Ji^1^, Xiaoning Zhang^1*^, Yong Zhu^1^, Michael L. Norton^2^, Lunfu Shen^3^, Wenhui Tan^3^, Xi Zheng^1^, Shuo Li^3^**

^1^ State Key Laboratory of Silkworm Genome Biology, College of Sericulture, Textile and Biomass Sciences, Southwest University, Chongqing 400715, China

^2^ Department of Chemistry, Marshall University, Huntington, WV 25755, United States

^3^ Chongqing Sericulture Science and Technology Research Institute, Chongqing Sericulture Technology Extension Station, Chongqing 400700, China

*** Correspondence:**Xiaoning Zhang
[xzhang@swu.edu.cn](mailto:xzhang@swu.edu.cn)

**Keywords: Silkworms, modified mulberry leaves, carbonized silk, molybdenum dioxide, supercapacitors.**

This paper is dedicated to the memory of Professor Yong Zhu, whose devotion to understanding the biology of *Bombyx mori* was unsurpassed.

**Table of Contents**

[1. Interpretation of abbreviations of different groups 3](#_Toc117168150)

[2. Assembly used to measure the electrical conductivity 3](#_Toc117168151)

[3. Preparation of the supercapacitor electrode 4](#_Toc117168152)

[4. Growth curves of silkworm larvae and their weights before cocooning 4](#_Toc117168153)

[5. Cocooning rates of silkworms 5](#_Toc117168154)

[6. Appearance of cocoons from the Mo-5 g/L and Mo-10 g/L groups 6](#_Toc117168155)

[7. Morphology of the silk gland 6](#_Toc117168156)

[8. Mo content determination 7](#_Toc117168157)

[9. Average width of the degummed silk fibers 8](#_Toc117168158)

[10. Results of EDS analysis of the degummed silk fibers 8](#_Toc117168159)

[11. Deconvolution of FTIR spectra 9](#_Toc117168160)

[12. Proposed conformational transition of silk fibroin under AMT feeding 10](#_Toc117168161)

[13. Representative optical microscopy images of the silk threads 12](#_Toc117168162)

[14. Average width of silk threads 12](#_Toc117168163)

[15. Mechanical properties of silk threads derived from each group 13](#_Toc117168164)

[16. Characterization of nanoparticles grown within CSF 17](#_Toc117168165)

[17. Deconvolution of Raman spectra 18](#_Toc117168166)

[18. Electrical conductivity test results 18](#_Toc117168167)

[19. The specific capacitance of the CSF electrodes 19](#_Toc117168168)

[20. Coulombic efficiency analysis 19](#_Toc117168169)

# 1. Interpretation of abbreviations of different groups

Table S1 Interpretation of abbreviations of different groups.

| **Groups** | **Interpretation** |
| --- | --- |
| Control | Silkworms were fed untreated mulberry leaves |
| Mo-0.05g/L | Silkworms were fed mulberry leaves treated with aqueous solution of AMT containing 0.05 g/L of Mo |
| Mo-0.1 g/L | Silkworms were fed mulberry leaves treated with aqueous solution of AMT containing 0.1 g/L of Mo |
| Mo-0.5 g/L | Silkworms were fed mulberry leaves treated with aqueous solution of AMT containing 0.5 g/L of Mo |
| Mo-1 g/L | Silkworms were fed mulberry leaves treated with aqueous solution of AMT containing 1 g/L of Mo |
| Mo-5 g/L | Silkworms were fed mulberry leaves treated with aqueous solution of AMT containing 5 g/L of Mo |
| Mo-10 g/L | Silkworms were fed mulberry leaves treated with aqueous solution of AMT containing 10 g/L of Mo |

# 2. Assembly used to measure the electrical conductivity


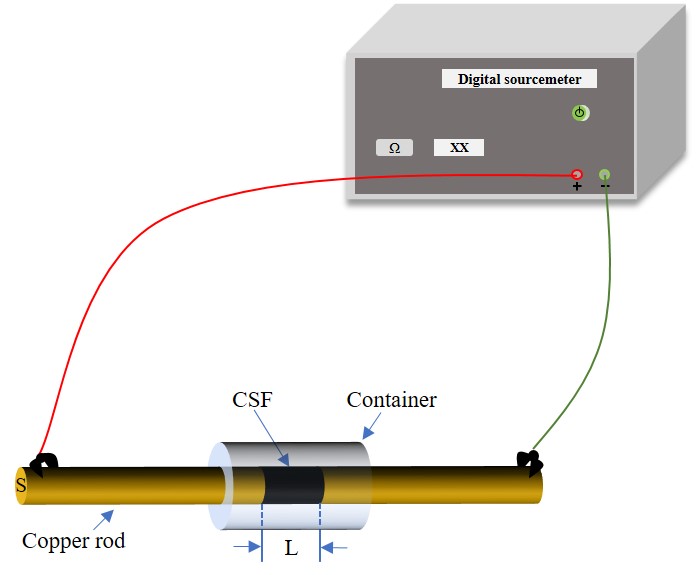


Figure S1. Device used to measure the electrical conductivity of CSF.

# 3. Preparation of the supercapacitor electrode

The nickel-foam pads (1×2 cm^2^) were thoroughly rinsed with water, followed by three ethanol rinses. The weight of the Ni foam was measured and recorded as *m_1_*. Then, the CSFs were cut into fine pieces and mixed with acetylene black and polytetrafluoroethylene (PTFE) in a mass ratio of 8:1:1 using ethanol as the solvent. The mixture was then pasted on the surface of the nickel-foam substrate in an area of 1×1 cm^2^, which was placed in an oven at 80 °C for 12 h. Subsequently, the two strips of nickel foam were combined to form a single piece by compression at a pressure of 10 MPa for 10 min. The weight of each piece was measured and recorded as *m_2_* (Figure S2). The mass of each sample (M) was calculated using the following equation:

*M* = (*m_2_* - *m_1_*) × 80% (1)


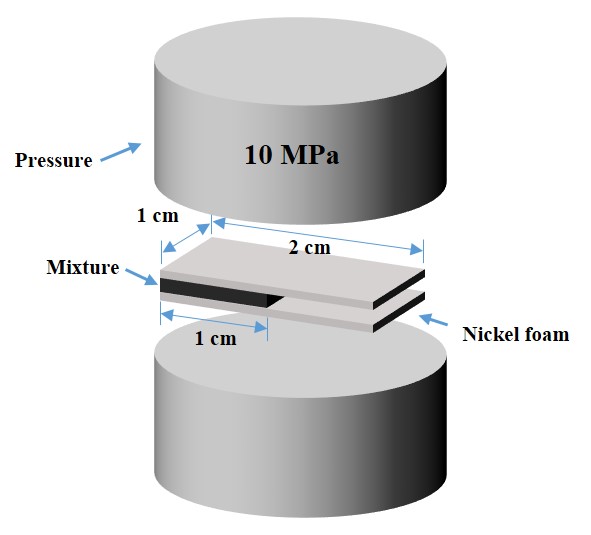


Figure S2. Schematic of the preparation of the working electrode.

# 4. Growth curves of silkworm larvae and their weights before cocooning

From the first day to the eighth day (i.e., immediately before cocooning) of the fifth instar, the weights of the larvae from each group were measured and recorded (Figure S3a). The curves in Figure S3a can be divided into two stages. The growth phase ranged from the first to the sixth day, and the stationary phase included days seven and eight. It can be observed that the weights of the larvae tended to decrease as the AMT feeding dosage increased. It is known that silkworms stop eating and excrete their intestinal content before cocooning (Sun et al., 2016); therefore, the weight measured at that time can be considered the most accurate. These data are shown in Figure S3b: the statistical analysis reveals that feeding dosages below 0.5 g/L of Mo did not obviously influence the body weights of the silkworms, but the weights of the silkworms decreased slightly when the Mo feeding dosage was 0.5 g/L and continued to decline with increasing dosage.


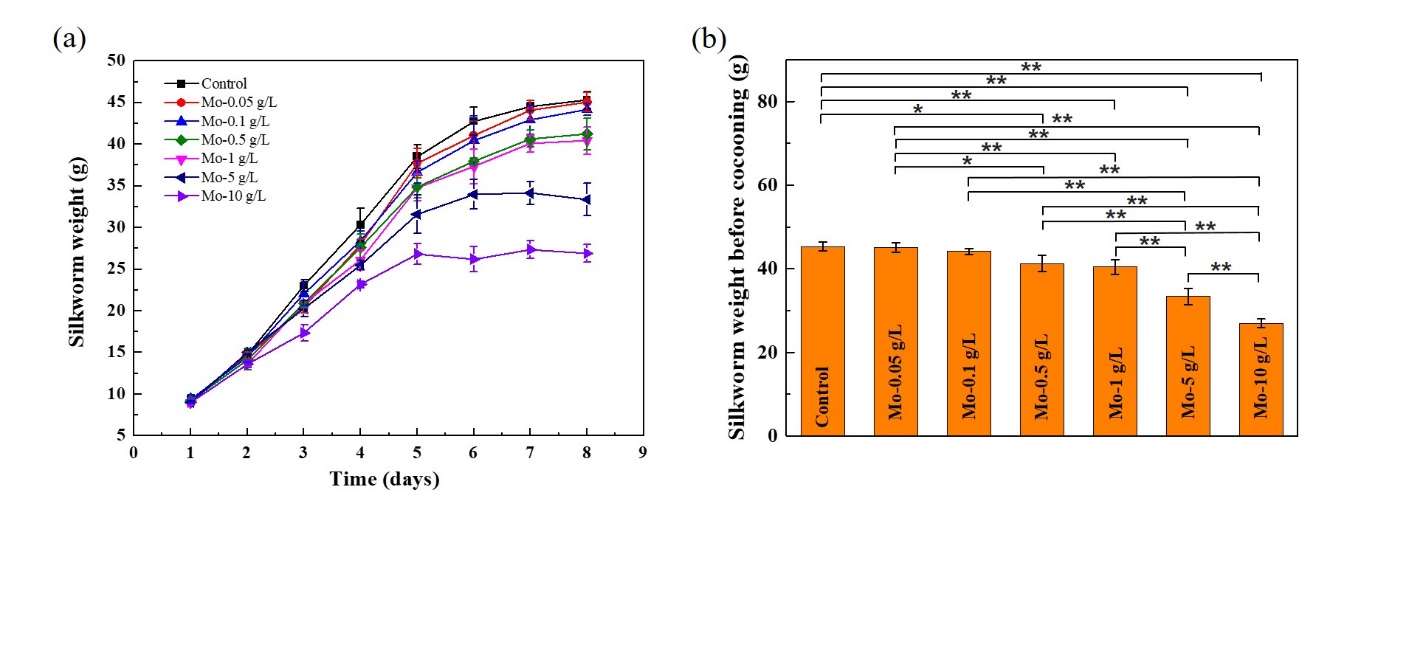


Figure S3. Effect of AMT feeding on the growth of silkworm larvae. (a) Growth curves of silkworm larvae from each group, and (b) the corresponding body weights measured before cocooning. The statistical analysis was performed using an unpaired, two-tailed t-test (***** P＜0.05, ****** P＜0.01, n = 3).

# 5. Cocooning rates of silkworms

The cocooning rates for both the Mo-5 g/L and Mo-10 g/L groups were too low to be used for practical analysis.


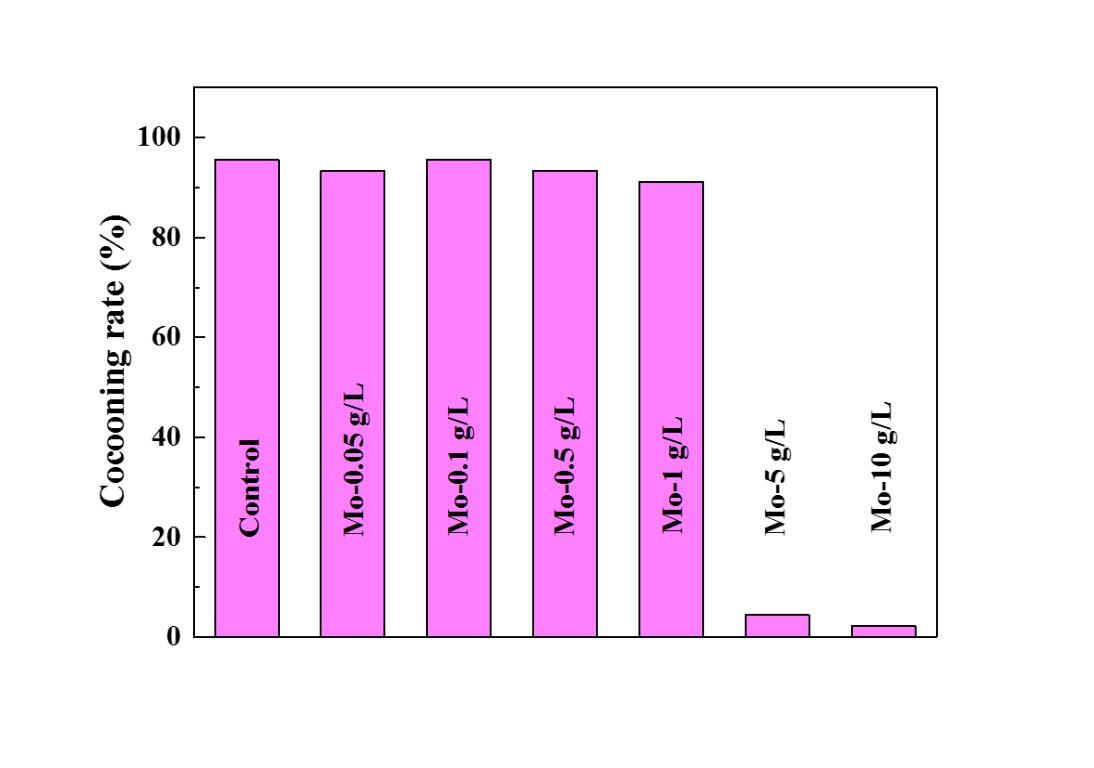


Figure S4. Cocooning rates of silkworms from each group.

# 6. Appearance of cocoons from the Mo-5 g/L and Mo-10 g/L groups

Cocoons from both the Mo-5 g/L and Mo-10 g/L groups appeared abnormal.


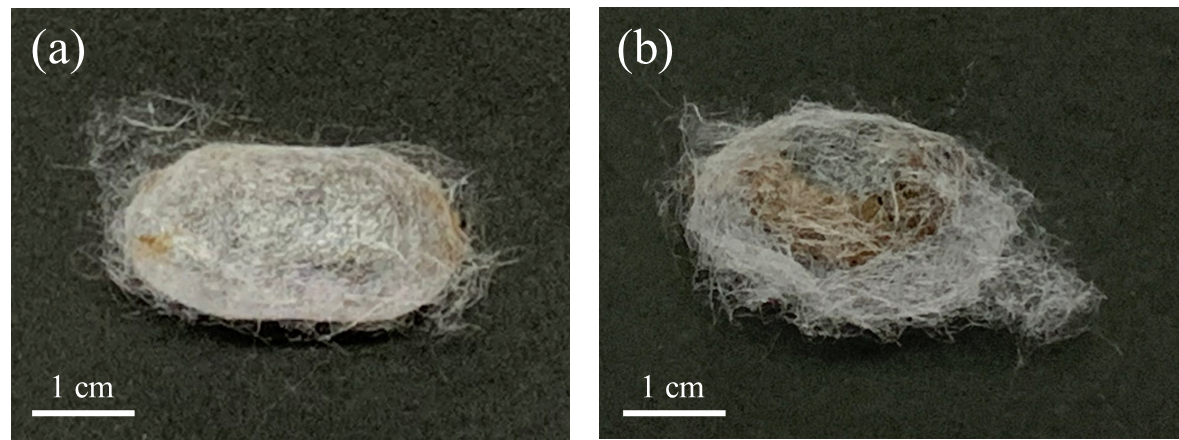


Figure S5. Representative images of cocoons from (a) Mo-5 g/L and (b) Mo-10 g/L groups.

# 7. Morphology of the silk gland

The silk gland of the silkworm is a highly specialized organ for the synthesis of silk protein and plays an important role in determining the properties of silk fibers (Chen et al., 2020). Intact silk glands from silkworms in each group were dissected on day 8 of the fifth-instar larvae and were found to have similar anatomical structures, but with variable sizes (Figure S6). It can be seen that the silk glands of each part gradually diminished in size with increasing AMT feeding dosage. This observation is consistent with the results of the cocoon size and cocoon shell ratio presented in Figure 3.


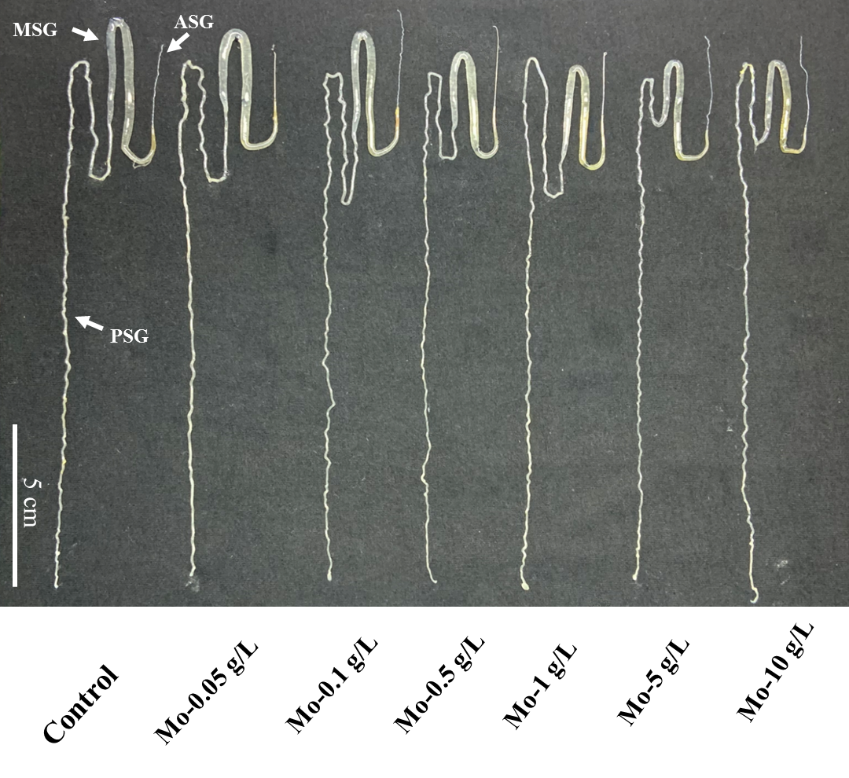


Figure S6. Dissected silk glands of silkworms from each group. ASG: anterior silk gland; MSG: middle silk gland; and PSG: posterior silk gland.

# 8. Mo content determination

ICP-OES was used to detect the content of elemental Mo incorporated within the degummed silk and the cocoons. It was found that the Mo content increased as the AMT feeding dose increased.

Table S2 Mo content in degummed silk.

| **Sample** | **Mo content (mg/kg)** |
| --- | --- |
| Control | 0.07±0.01 |
| Mo-0.05 g/L | 0.26±0.02 |
| Mo-0.1 g/L | 0.54±0.03 |
| Mo-0.5 g/L | 1.43±0.01 |
| Mo-1 g/L | 2.34±0.06 |

Table S3 Mo content in the cocoons.

| **Sample** | **Mo content (mg/kg)** |
| --- | --- |
| Control | 0.98±0.05 |
| Mo-0.05 g/L | 1.34±0.13 |
| Mo-0.1 g/L | 1.47±0.02 |
| Mo-0.5 g/L | 2.99±0.06 |
| Mo-1 g/L | 4.92±0.12 |

# 9. Average width of the degummed silk fibers


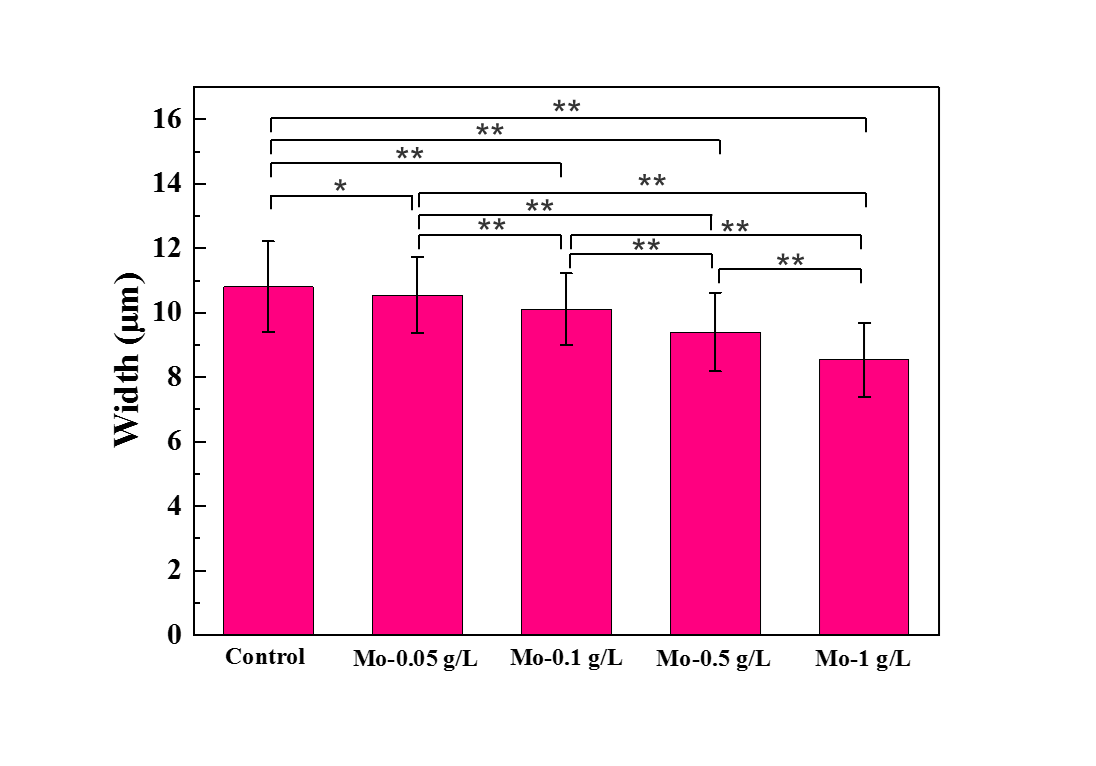


Figure S7. Effect of AMT on the width of the SF fibers. (***** P＜0.05, ****** P＜0.01, n = 300).

# 10. Results of EDS analysis of the degummed silk fibers

Figures S8 and S9 present the EDS elemental mapping images and EDS spectra of the degummed silk fibers in the control and Mo-1 g/L groups, respectively. It can be seen that Mo was distributed throughout the silk fiber of the Mo-1 g/L group, while the Mo content in the silk of the control group was below the detection limit of EDS.

**
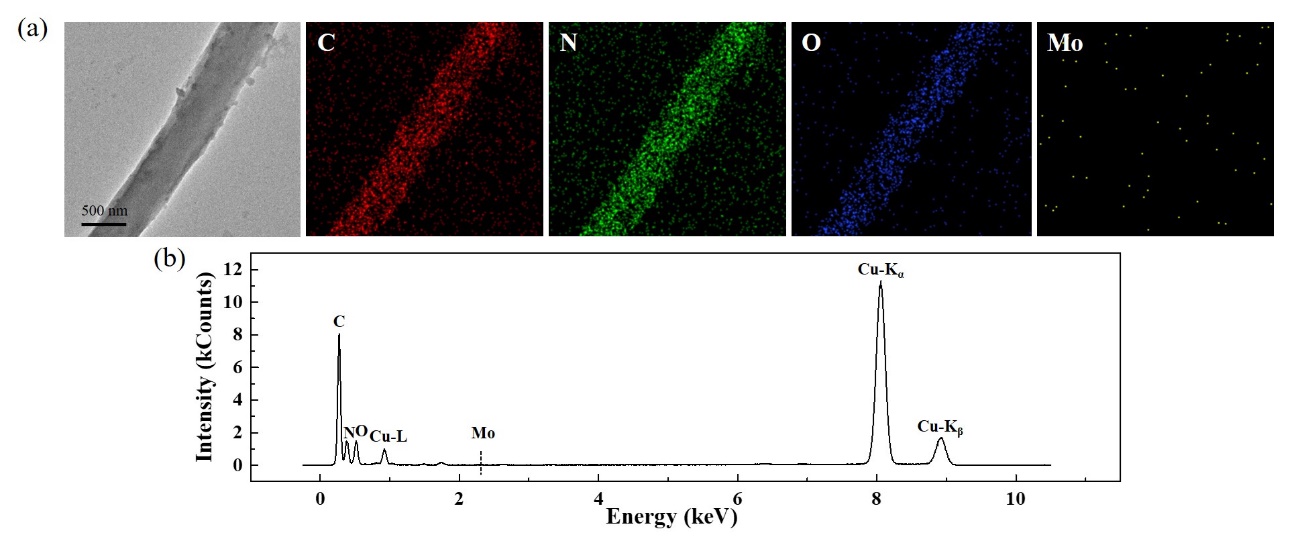
**

Figure S8. Sample EDS analysis of a silk fiber from the control group. (a) EDS element mapping images showing the C, N, O and Mo distributions, and (b) the corresponding EDS spectrum.


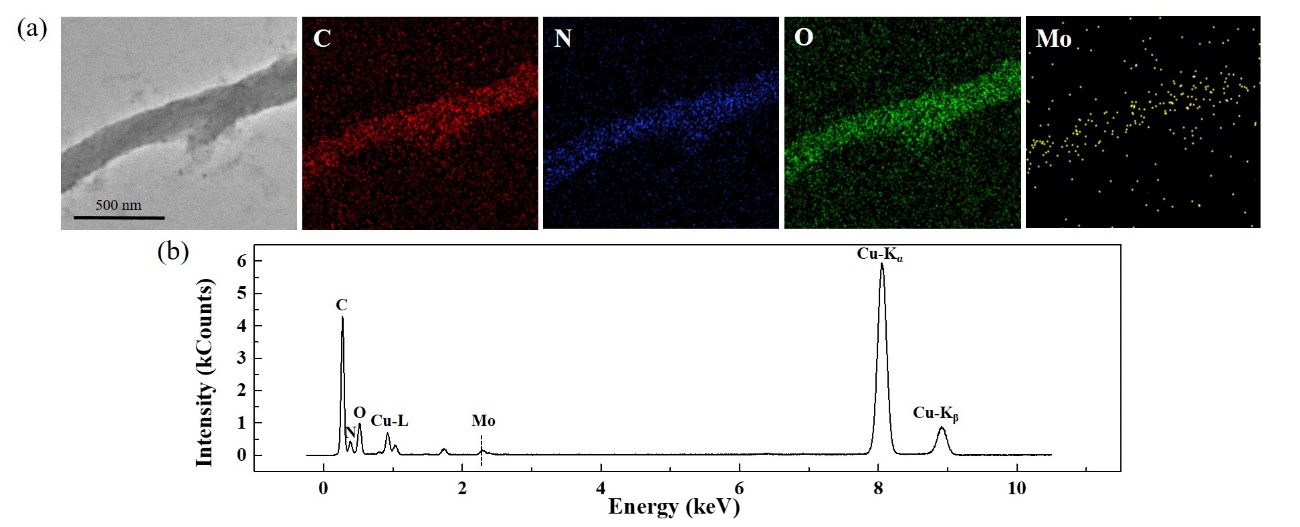


Figure S9. Sample EDS analysis of a silk fiber from the Mo-1 g/L group. (a) EDS element mapping images showing the C, N, O and Mo distributions, and (b) the corresponding EDS spectrum.

# 11. Deconvolution of FTIR spectra

Deconvolution of the silk amide I band was performed using a Gaussian fitting function (Artali et al., 2008). The peaks centered at 1634 cm^–1^, 1673 cm^–1^, and 1706 cm^–1^ were assigned to *β*-sheets, random coils/*α*-helixes, and *β*-turns, respectively (Cheng et al., 2017; Yan et al., 2014).


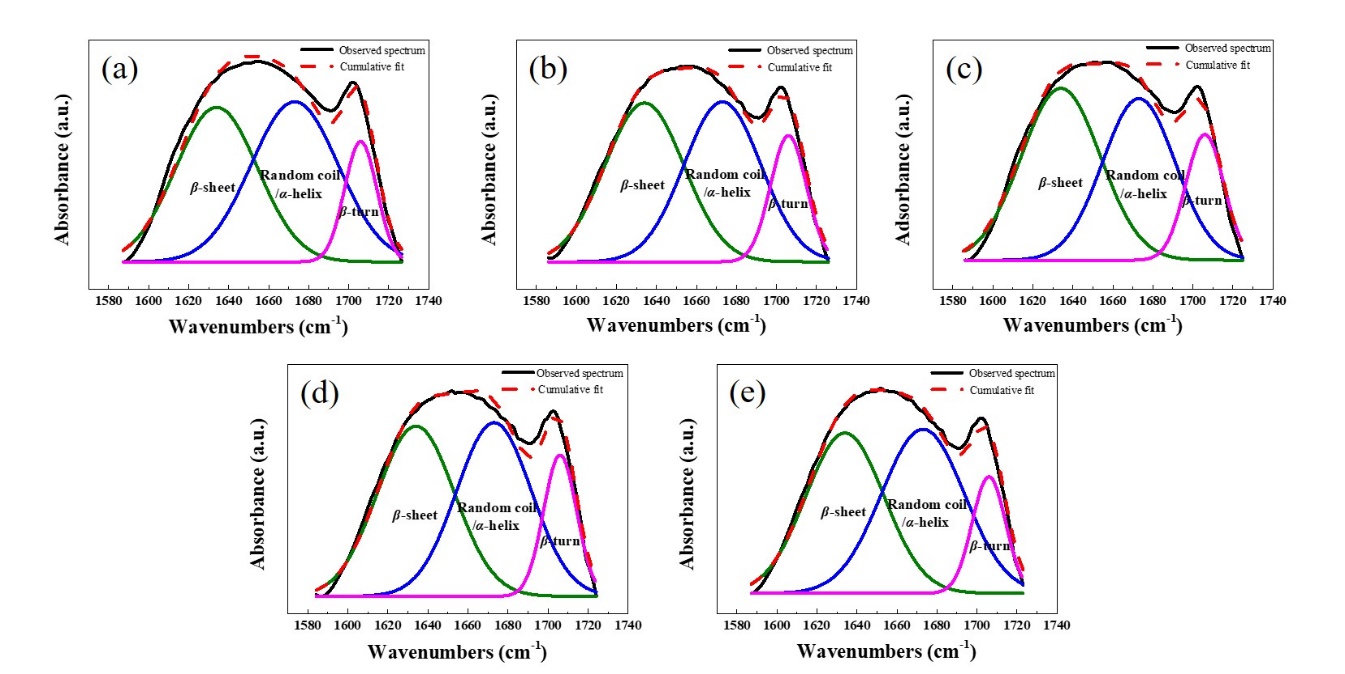


Figure S10. Representative graphs showing the deconvolution of the silk amide I band into its structural components. The colored lines represent the fitted peaks of the components

An analysis and comparison of each deconvoluted component in the sample groups are presented in Table S4.

Table S4 Population of each deconvoluted component.

| **Structure**  **Group** | ***β*-sheet** | **Random coil/ *α*-helix** | | ***β*-turn** |
| --- | --- | --- | --- | --- |
| Control | 40.82%±0.65% | 45.77%±0.43% | | 13.41%±0.82% |
| Mo-0.05 g/L | 43.17%±0.99% | 41.87%±0.11% | | 14.97%±1.10% |
| Mo-0.1 g/L | 44.70%±0.89% | 38.41%±0.54% | | 16.89%±1.32% |
| Mo-0.5 g/L | 41.67%±1.07% | 42.56%±0.46% | | 15.77%±0.62% |
| Mo-1 g/L | 41.17%±1.02% | | 45.85%±1.31% | 12.99%±0.32% |

# 12. Proposed conformational transition of silk fibroin under AMT feeding

Previous studies have confirmed that metallic ions such as K^+^ (Ruan et al., 2007), Ca^2^^+^ (Zhou et al., 2005) and Cu^2+^ (Li et al., 2003)influence the conformational transitions of silk proteins by binding them and interfering with the interactions among the silk fibrils (Zhang et al., 2012). It is known that the natural silk-spinning process of the liquid-to-solid transition involves a conformational change of the silk fibroin from a random coil/α-helix into a *β*-sheet secondary structure (Zhou et al., 2005). We assume that the molybdate ions bind to the silk proteins, acting as the core structure and inducing conformational transitions of silk fibroin from random/α-helix into *β*-sheet structures at Mo feeding dosages of less than 0.1 g/L (Wang et al., 2016; Xu et al., 2008). However, as the Mo feeding dosage increased from 0.1 g/L to 1 g/L, the excess molybdate bound to the silk proteins led to repulsion, thereby inhibiting the conformational transition of random/α-helix silk fibroin to *β*-sheets (Zhang et al., 2012).


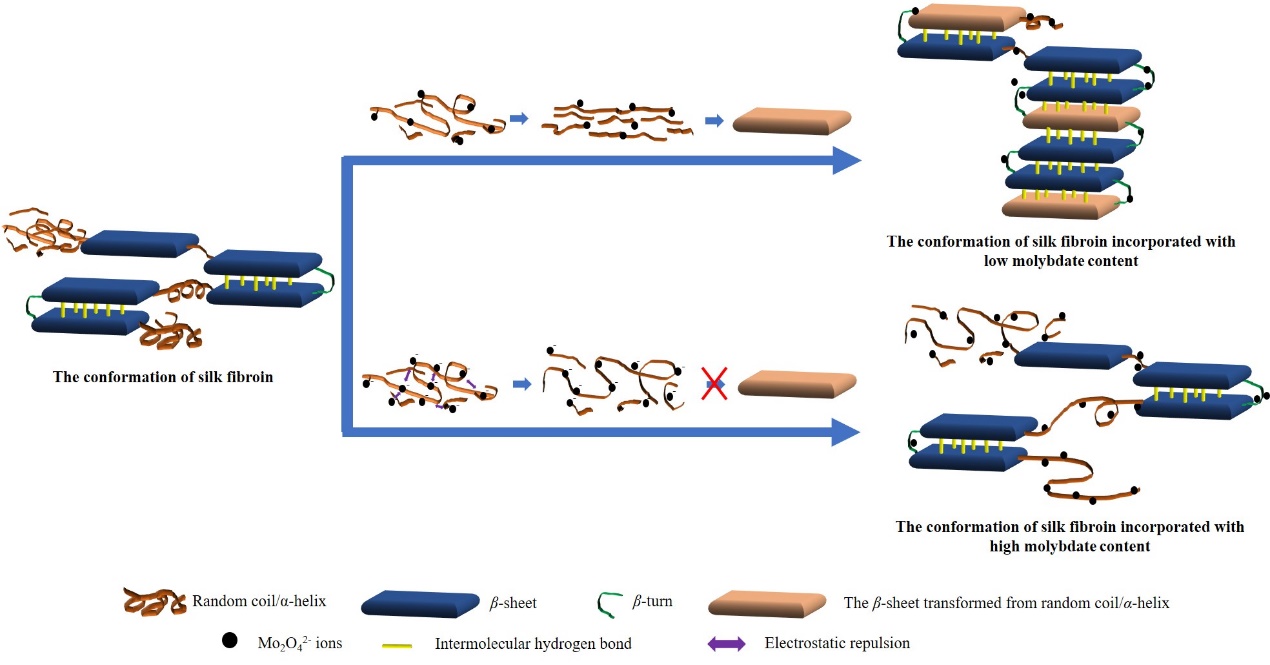


Figure S11. Schematic diagram of the proposed mechanism for the conformational changes of silk fibroin.

# 13. Representative optical microscopy images of the silk threads


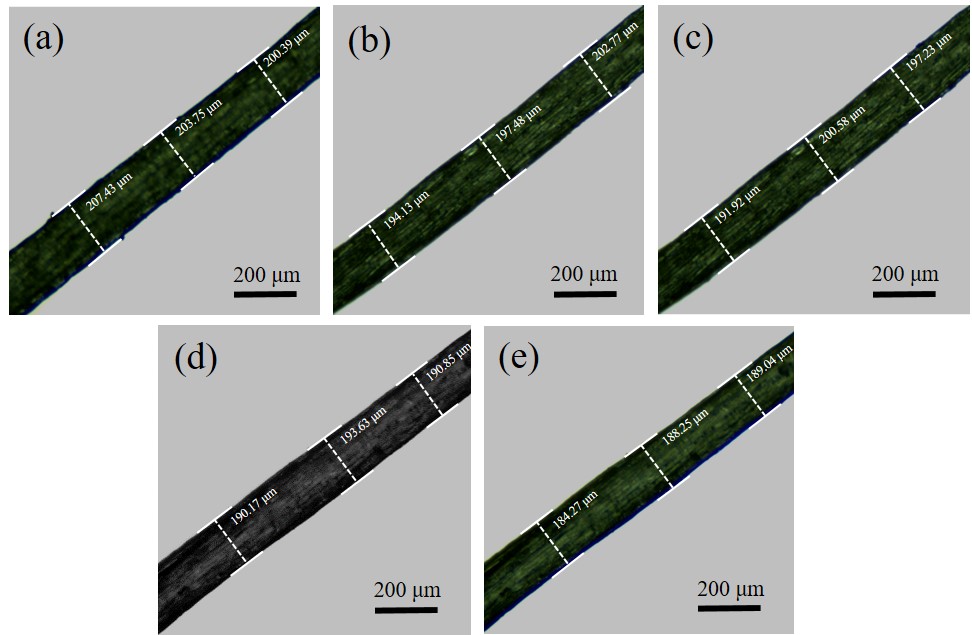


Figure S12. Representative optical microscopy images of silk threads composed of 100 single silk fibers without a hollow region: (a) control group, (b) Mo-0.05 g/L group, (c) Mo-0.01 g/L group, (d) Mo-0.5 g/L group, and (e) Mo-1 g/L group.

# 14. Average width of silk threads


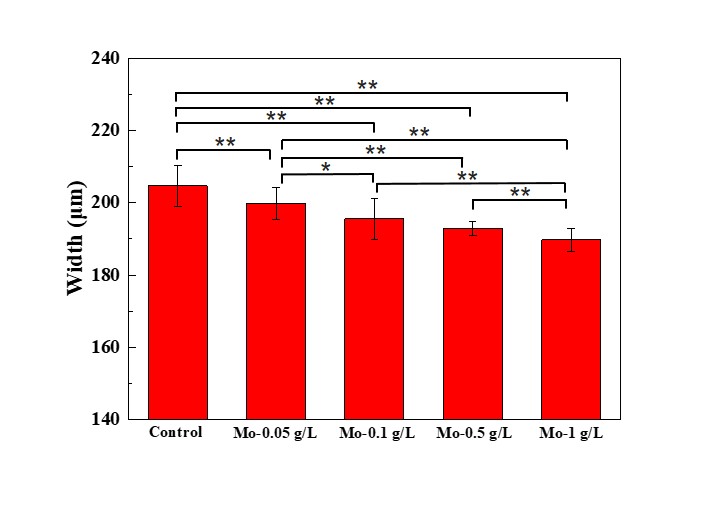


Figure S13. Average width of silk threads used for tensile testing.

# 15. Mechanical properties of silk threads derived from each group

Table S5 Mechanical properties and widths of silk threads derived from the control group.

| **Number** | **Fracture load**  **[cN]** | **Strain**  **[%]** | **Width**  **[*μ*m]** | **Fracture strength**  **[GPa]** | **Toughness modulus**  **[GJ/m^3^]** |
| --- | --- | --- | --- | --- | --- |
| 1 | 1266.00 | 42.90 | 190.59 | 443.97 | 13.62 |
| 2 | 1409.00 | 43.20 | 206.28 | 421.81 | 12.19 |
| 3 | 1399.00 | 44.00 | 206.84 | 416.56 | 12.39 |
| 4 | 1541.00 | 43.40 | 208.32 | 452.36 | 11.77 |
| 5 | 1573.00 | 45.20 | 200.48 | 498.56 | 14.84 |
| 6 | 1493.00 | 43.10 | 214.05 | 415.09 | 11.97 |
| 7 | 1402.00 | 37.60 | 214.39 | 388.57 | 9.70 |
| 8 | 1318.00 | 38.70 | 198.22 | 427.32 | 11.09 |
| 9 | 1368.00 | 41.60 | 209.08 | 398.65 | 11.41 |
| 10 | 1411.00 | 51.20 | 208.81 | 412.24 | 14.39 |
| 11 | 1106.00 | 48.00 | 208.66 | 323.60 | 10.50 |
| 12 | 1454.00 | 40.50 | 202.47 | 451.83 | 11.91 |
| 13 | 1318.00 | 45.00 | 204.32 | 402.18 | 12.62 |
| 14 | 1363.00 | 44.00 | 203.54 | 419.11 | 12.53 |
| 15 | 1415.00 | 35.50 | 202.11 | 441.28 | 10.38 |
| 16 | 1429.00 | 35.10 | 201.17 | 449.82 | 10.48 |
| 17 | 1253.00 | 35.20 | 202.74 | 388.33 | 8.83 |
| 18 | 1255.00 | 42.80 | 202.84 | 388.57 | 11.81 |
| Average | 1376.28 | 42.06 | 204.72 | 418.88 | 11.80 |
| Standard deviation | 112.56 | 4.36 | 5.66 | 37.04 | 1.54 |

Table S6 Mechanical properties and widths of silk threads derived from the Mo-0.05 g/L group.

| **Number** | **Fracture load**  **[cN]** | **Strain**  **[%]** | **Width**  **[*μ*m]** | **Fracture strength**  **[GPa]** | **Toughness modulus**  **[GJ/m^3^]** |
| --- | --- | --- | --- | --- | --- |
| 1 | 1327.00 | 47.40 | 196.45 | 438.04 | 14.59 |
| 2 | 1231.00 | 40.20 | 197.50 | 402.04 | 10.54 |
| 3 | 1409.00 | 55.10 | 193.68 | 478.49 | 18.73 |
| 4 | 1516.00 | 40.80 | 199.26 | 486.42 | 13.57 |
| 5 | 1674.00 | 41.50 | 197.38 | 547.37 | 15.09 |
| 6 | 1421.00 | 36.10 | 199.79 | 453.49 | 10.99 |
| 7 | 1338.00 | 45.60 | 195.81 | 444.56 | 13.72 |
| 8 | 1483.00 | 45.10 | 199.14 | 476.36 | 15.03 |
| 9 | 1429.00 | 46.30 | 208.14 | 420.21 | 12.85 |
| 10 | 1484.00 | 44.50 | 201.74 | 464.50 | 14.38 |
| 11 | 1512.00 | 42.60 | 202.64 | 469.08 | 13.05 |
| 12 | 1497.00 | 47.80 | 195.29 | 500.02 | 16.41 |
| 13 | 1536.00 | 40.90 | 201.50 | 481.90 | 13.32 |
| 14 | 1517.00 | 43.90 | 197.60 | 494.92 | 14.79 |
| 15 | 1454.00 | 45.00 | 193.80 | 493.15 | 15.33 |
| 16 | 1490.00 | 32.70 | 208.81 | 435.32 | 8.96 |
| 17 | 1543.00 | 51.00 | 205.53 | 465.33 | 17.26 |
| 18 | 1545.00 | 50.10 | 202.89 | 478.12 | 16.89 |
| Average | 1467.00 | 44.26 | 199.83 | 468.29 | 14.20 |
| Standard deviation | 99.23 | 5.30 | 4.51 | 33.32 | 2.44 |

Table S7 Mechanical properties and widths of silk threads derived from the Mo-0.1 g/L group.

| **Number** | **Fracture load**  **[cN]** | **Strain**  **[%]** | **Width**  **[*μ*m]** | **Fracture strength**  **[GPa]** | **Toughness modulus**  **[GJ/m^3^]** |
| --- | --- | --- | --- | --- | --- |
| 1 | 1495.00 | 45.00 | 192.61 | 513.36 | 15.47 |
| 2 | 1417.00 | 39.00 | 199.46 | 453.71 | 11.83 |
| 3 | 1514.00 | 53.00 | 199.14 | 486.35 | 18.36 |
| 4 | 1417.00 | 52.40 | 195.55 | 472.03 | 13.75 |
| 5 | 1512.00 | 44.10 | 199.23 | 485.24 | 15.37 |
| 6 | 1451.00 | 45.20 | 187.67 | 524.81 | 16.08 |
| 7 | 1558.00 | 43.50 | 183.91 | 586.77 | 18.54 |
| 8 | 1423.00 | 49.90 | 203.41 | 438.10 | 15.29 |
| 9 | 1518.00 | 46.50 | 191.88 | 525.21 | 16.99 |
| 10 | 1590.00 | 51.90 | 201.15 | 500.62 | 18.09 |
| 11 | 1695.00 | 42.80 | 197.15 | 555.52 | 15.52 |
| 12 | 1551.00 | 52.50 | 193.14 | 529.64 | 18.80 |
| 13 | 1520.00 | 43.60 | 193.71 | 516.01 | 14.61 |
| 14 | 1544.00 | 41.80 | 187.57 | 559.03 | 16.08 |
| 15 | 1539.00 | 46.20 | 192.05 | 531.55 | 16.61 |
| 16 | 1580.00 | 40.50 | 201.61 | 495.19 | 14.22 |
| 17 | 1559.00 | 44.90 | 204.17 | 476.40 | 15.41 |
| 18 | 1521.00 | 49.10 | 196.33 | 502.69 | 17.28 |
| Average | 1522.44 | 46.22 | 195.54 | 508.46 | 16.02 |
| Standard deviation | 68.60 | 4.32 | 5.71 | 37.66 | 1.83 |

Table S8 Mechanical properties and widths of silk threads derived from the Mo-0.5 g/L group.

| **Number** | **Fracture load**  **[cN]** | **Strain**  **[%]** | **Width**  **[*μ*m]** | **Fracture strength**  **[GPa]** | **Toughness modulus**  **[GJ/m^3^]** |
| --- | --- | --- | --- | --- | --- |
| 1 | 1330.00 | 45.70 | 193.66 | 451.77 | 13.20 |
| 2 | 1408.00 | 36.10 | 190.92 | 492.08 | 11.63 |
| 3 | 1414.00 | 34.30 | 188.96 | 504.49 | 11.19 |
| 4 | 1333.00 | 45.40 | 197.13 | 437.00 | 13.86 |
| 5 | 1399.00 | 42.10 | 194.45 | 471.33 | 13.36 |
| 6 | 1223.00 | 41.80 | 193.13 | 417.71 | 11.59 |
| 7 | 1233.00 | 44.40 | 192.50 | 423.87 | 12.83 |
| 8 | 1231.00 | 42.70 | 193.17 | 420.25 | 12.32 |
| 9 | 1325.00 | 37.70 | 190.61 | 464.57 | 12.04 |
| 10 | 1389.00 | 35.70 | 196.77 | 457.01 | 10.96 |
| 11 | 1478.00 | 40.80 | 192.84 | 506.31 | 14.28 |
| 12 | 1399.00 | 39.40 | 192.95 | 478.69 | 12.99 |
| 13 | 1305.00 | 43.70 | 192.24 | 449.85 | 13.07 |
| 14 | 1378.00 | 39.30 | 191.06 | 480.88 | 12.37 |
| 15 | 1324.00 | 40.30 | 193.13 | 452.19 | 11.38 |
| 16 | 1397.00 | 42.40 | 192.74 | 479.05 | 12.97 |
| 17 | 1370.00 | 43.70 | 194.70 | 460.38 | 12.97 |
| 18 | 1210.00 | 47.10 | 191.56 | 420.05 | 13.33 |
| Average | 1341.44 | 41.26 | 192.92 | 459.30 | 12.57 |
| Standard deviation | 76.63 | 3.63 | 2.03 | 28.16 | 0.94 |

Table S9 Mechanical properties and widths of silk threads derived from the Mo-1 g/L group.

| **Number** | **Fracture load**  **[cN]** | **Strain**  **[%]** | **Width**  **[*μ*m]** | **Fracture strength**  **[GPa]** | **Toughness modulus**  **[GJ/m^3^]** |
| --- | --- | --- | --- | --- | --- |
| 1 | 1185.00 | 34.80 | 184.88 | 441.66 | 10.28 |
| 2 | 1238.00 | 39.00 | 192.36 | 426.20 | 10.95 |
| 3 | 1177.00 | 36.80 | 192.64 | 404.04 | 9.89 |
| 4 | 1155.00 | 36.60 | 192.81 | 395.78 | 9.93 |
| 5 | 1361.00 | 34.00 | 192.72 | 466.80 | 10.62 |
| 6 | 1134.00 | 38.80 | 186.73 | 414.31 | 10.68 |
| 7 | 1145.00 | 36.10 | 193.27 | 390.48 | 9.10 |
| 8 | 1227.00 | 37.50 | 195.58 | 408.63 | 10.26 |
| 9 | 1030.00 | 32.40 | 192.89 | 352.64 | 7.86 |
| 10 | 1079.00 | 36.70 | 187.13 | 392.52 | 9.66 |
| 11 | 1076.00 | 31.20 | 189.42 | 382.02 | 8.21 |
| 12 | 1100.00 | 31.50 | 189.25 | 391.25 | 8.17 |
| 13 | 1116.00 | 43.60 | 186.61 | 408.25 | 12.25 |
| 14 | 1191.00 | 37.70 | 187.24 | 432.76 | 10.67 |
| 15 | 1131.00 | 41.20 | 186.69 | 413.38 | 11.33 |
| 16 | 1022.00 | 40.40 | 189.10 | 364.08 | 9.48 |
| 17 | 898.00 | 39.40 | 188.98 | 320.31 | 8.88 |
| 18 | 1070.00 | 42.30 | 187.10 | 389.37 | 10.85 |
| Average | 1129.72 | 37.22 | 189.74 | 399.69 | 9.95 |
| Standard deviation | 99.99 | 3.55 | 3.10 | 33.48 | 1.18 |

# 16. Characterization of nanoparticles grown within CSF

Figure S14 confirms that the nanoscale particles were embedded in the CSF. The lattice-resolved HRTEM image reveals an interplanar spacing of 0.23 nm, corresponding to the (021) plane of MoO_2_. Furthermore, the fast Fourier transform (FFT) spots indicated that the (021) plane of MoO_2_ was present.


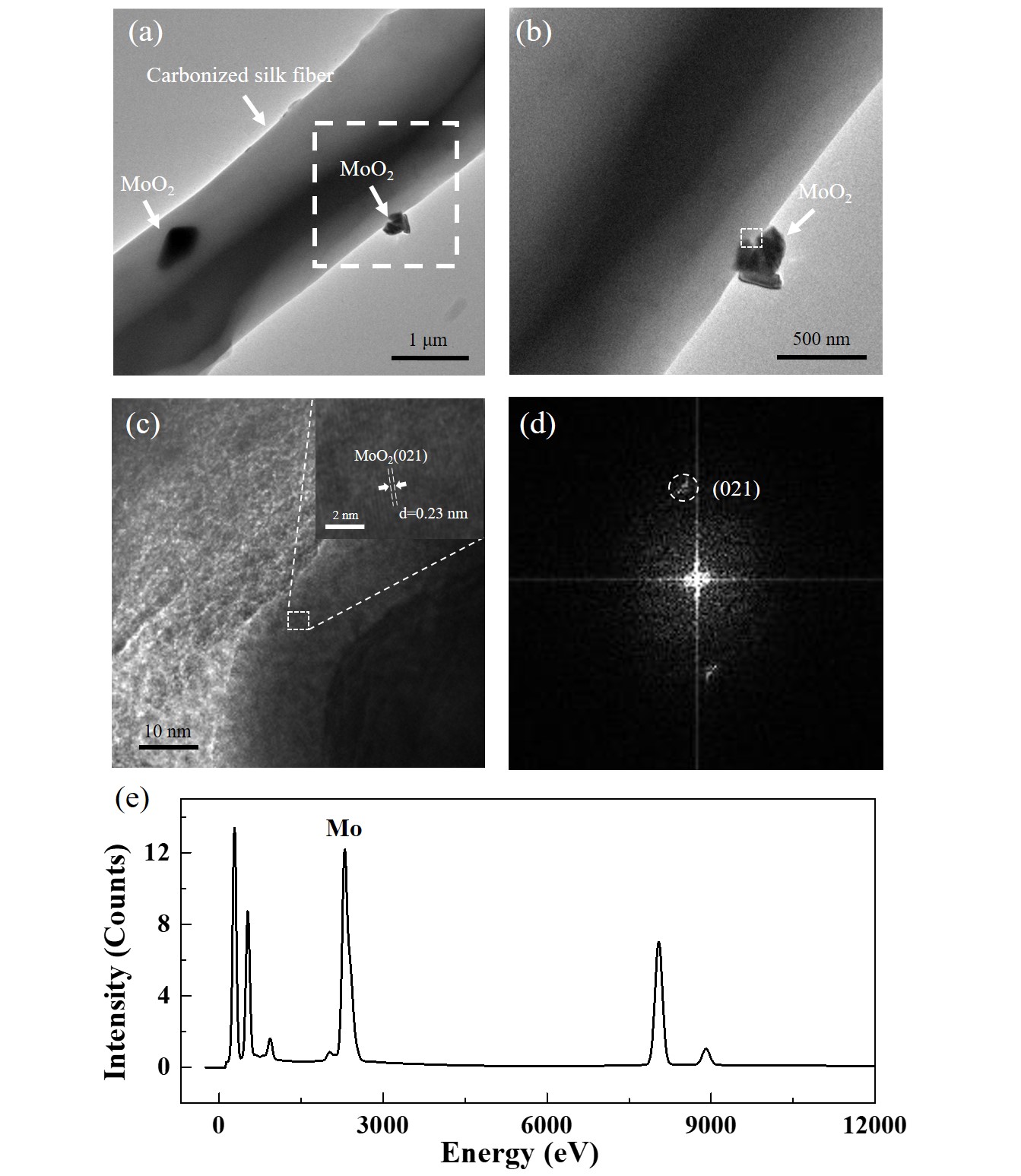


Figure S14. TEM imaging and corresponding analysis of CSF and the nanoparticle incorporated. (a) TEM image of CSF derived from Mo-1 g/L group, (b) magnified TEM image of the dashed-line region in Figure S14a, (c) magnified image of the dashed box in (b), where the inset shows the lattice-resolved HRTEM image; (d) FFT pattern corresponding to the region circled by the dashed line; and (e) EDS spectrum of the observed nanoparticle in the carbonized silk.

# 17. Deconvolution of Raman spectra


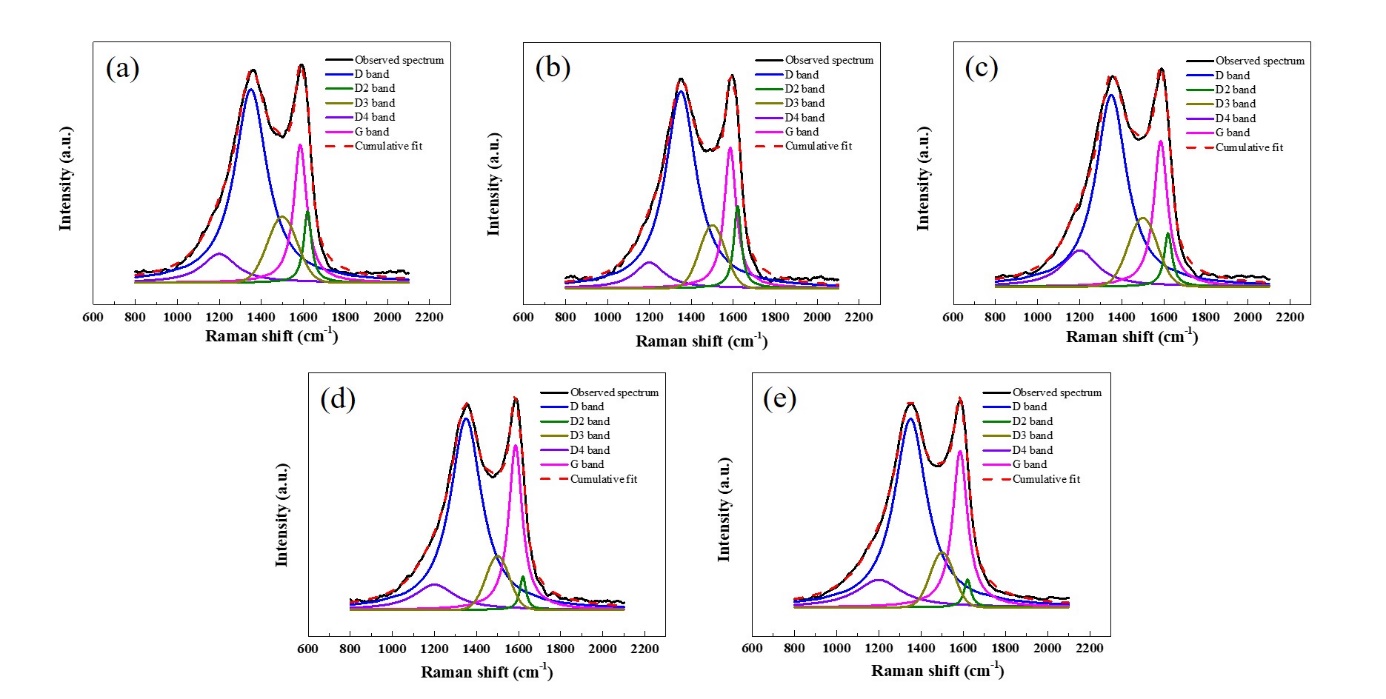


Figure S15. Representative Raman spectra and their deconvolution into D, D2, D3, D4, and G bands: (a) control group, (b) Mo-0.05 g/L group, (c) Mo-0.01 g/L group, (d) Mo-0.5 g/L group, and (e) Mo-1 g/L group.

# 18. Electrical conductivity test results

Table S10 Electrical conductivity test results.

| **Groups** | **Electrical conductivity (S/m)** |
| --- | --- |
| Control | 54.35±4.59 |
| Mo-0.05 g/L | 52.96±4.63 |
| Mo-0.1 g/L | 54.72±3.82 |
| Mo-0.5 g/L | 69.97±6.57 |
| Mo-1 g/L | 88.27±2.54 |

# 19. The specific capacitance of the CSF electrodes


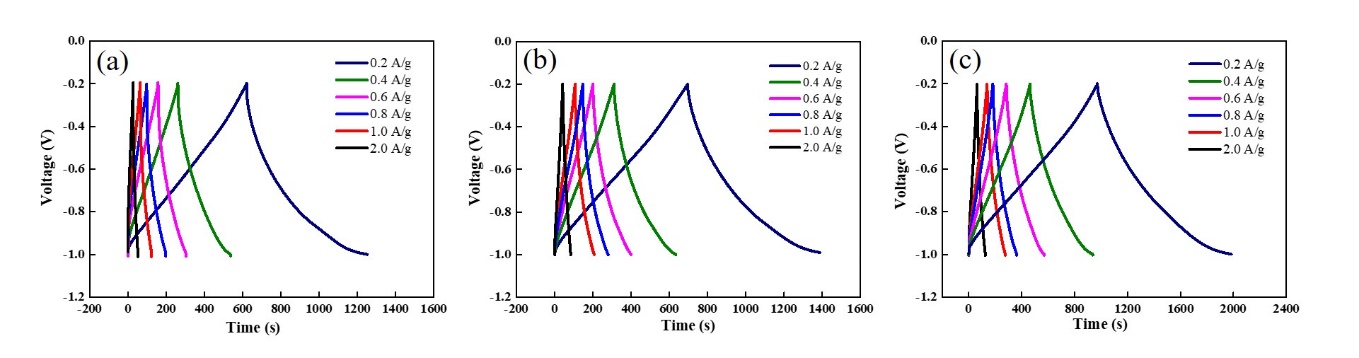


Figure S16. The GCD curves of the CSF electrodes prepared from (a) Mo-0.05 g/L group, (b) Mo-0.01 g/L group, (c) Mo-0.5 g/L group.

Table S11 Specific capacitance values of the CSF electrodes from all groups at different current densities

**Current**

**density**

| **Specific capacitance**  **Groups** | **0.2 A/g** | **0.4 A/g** | **0.6 A/g** | **0.8 A/g** | **1.0 A/g** | **2.0 A/g** |
| --- | --- | --- | --- | --- | --- | --- |
| Control | 102 F/g | 71 F/g | 62 F/g | 57 F/g | 51 F/g | 48 F/g |
| Mo-0.05 g/L | 126 F/g | 111 F/g | 103 F/g | 87 F/g | 74 F/g | 62 F/g |
| Mo-0.1 g/L | 167 F/g | 131 F/g | 122 F/g | 104 F/g | 98 F/g | 86 F/g |
| Mo-0.5 g/L | 203 F/g | 189 F/g | 171 F/g | 152 F/g | 140 F/g | 126 F/g |
| Mo-1 g/L | 298 F/g | 274 F/g | 258 F/g | 243 F/g | 236 F/g | 222 F/g |

# 20. Coulombic efficiency analysis

The Coulomb efficiency is the ratio of the charging time to the discharge time of the electrode material and reflects the reversibility and energy loss during the charge and discharge processes (Wang et al., 2017). As shown in Figure S17, the CSF electrode exhibited superior cyclic performance as an anode material for supercapacitors and delivered a Coulombic efficiency of 99.54% after 5000 cycles.


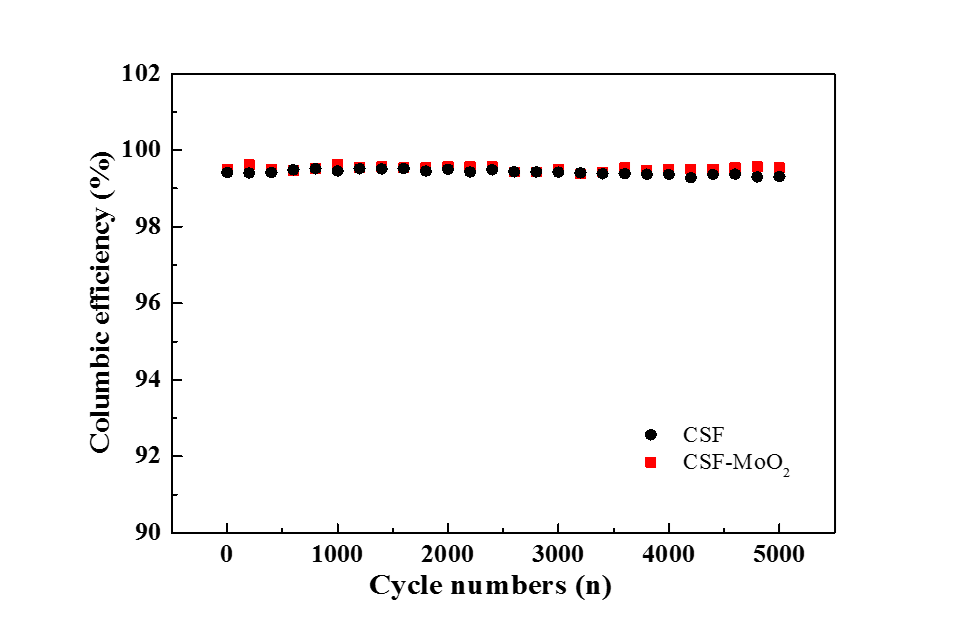


Figure S17. Coulombic efficiency of both the CSF and CSF-MoO_2_ electrodes at a current density of 2 A/g.

**Reference**

Chen, T., Sun, Q., Ma, Y., Zeng, W., Liu, R., Qu, D., Huang, L., Xu, H. (2020). A transcriptome atlas of silkworm silk glands revealed by PacBio single-molecule long-read sequencing. *Mol. Genet. Genomics*. 295, 1227-1237. doi:[10.1007/s00438-020-01691-9](https://doi.org/10.1007/s00438-020-01691-9).

Cheng, L., Huang, H., Chen, S. Wang, W., Dai, F., Zhao, H. (2017). Characterization of silkworm larvae growth and properties of silk fibers after direct feeding of copper or silver nanoparticles. *Mater. Des.* 129, 125-134. doi:[10.1016/j.matdes.2017.04.096](https://doi.org/10.1016/j.matdes.2017.04.096).

Li, J.Y., Yang, H.J., Lan, T.Y., Wei, H., Zhang, H.R., Chen, M., Fan, W., Ma, Y.Y., Xiong, B. (2011). Expression profiling and regulation of genes related to silkworm posterior silk gland development and fibroin synthesis. *J. Proteome Res.* 10, 3551-3564. doi:10.1021/pr200196x.

Liang, J., Zhang, X., Yan, C., Wang, Y., Norton, M.L., Wei, X., Donley, C., Zhu, Y., Xiao, P., Zhang, Y. (2020). Preparation and enhanced supercapacitance performance of carbonized silk by feeding silkworms MoO_2_ nanoparticles. *Mater. Des.* 196, 109137. doi:[10.1016/j.matdes.2020.109137](https://doi.org/10.1016/j.matdes.2020.109137).

Ruan, Q., Zhou, P., Hu, B., Ji, D. (2007). An investigation into the effect of potassium ions on the folding of silk fibroin studied by generalized two-dimensional NMR-NMR correlation and Raman spectroscopy. *FEBS J.* 275, 219-232. doi:[10.1111/j.1742-4658.2007.06191.x](https://doi.org/10.1111/j.1742-4658.2007.06191.x).

Wang, X., Li, Y., Liu, Q., Chen, Q., Xia, Q., Zhao, P. (2016). In vivo effects of metal ions on conformation and mechanical performance of silkworm silks. *Biochim. Biophys. Acta Biomembr.* 1861, 567-576. doi:[10.1016/j.bbagen.2016.11.025](https://doi.org/10.1016/j.bbagen.2016.11.025)

Wang, H., Liu, J., Chen, Z., Chen, S., Sum, T.C., Lin, J., Shen, Z.X. (2017). Synergistic capacitive behavior between polyaniline and carbon black. *Electrochim. Acta.* 230, 236-244. doi:[10.1016/j.electacta.2017.01.164](https://doi.org/10.1016/j.electacta.2017.01.164).

Xu, X., Zhang, B. (2008). The mechanism research of metal ions on the conformation transition of silk fibroin. *Chinese Polym. Bull.* 2008, 48-51.

Yan, C., Yang, B., Yu, Z. (2014). Methanol-induced conformation transition of gland fibroin monitored by FTIR spectroscopy and terahertz spectroscopy. *Analyst.* 139, 6310-6310. doi:[10.1016/10.1039/c3an01547e](https://doi.org/10.1016/10.1039/c3an01547e).

Zhang, Y., Jiang, T., Zheng, Y., Zhou, P. (2012). Interference of EGCG on the Zn(II)-induced conformational transition of silk fibroin as a model protein related to neurodegenerative diseases. *Soft Matter.* 8, 5543-5549. doi:[10.1039/C2SM25099C](https://doi.org/10.1039/C2SM25099C).

Zhou, L., Chen, X., Shao, Z., Huang, Y., Knight, D.P. (2005). Effect of metallic ions on silk formation in the mulberry silkworm, *Bombyx mori*. *J. Phys. Chem. B.* 109, 16937. doi:[10.1021/jp050883m](https://doi.org/10.1021/jp050883m).

Zhou, L., Chen, X., Shao, Z., Zhou, P., Knight, D.P., Fritz, V. (2003). Copper in the silk formation process of *Bombyx mori* silkworm. *FEBS Lett.* 554, 337-341. doi:[10.1016/S0014-5793(03)01184-0](https://doi.org/10.1016/S0014-5793(03)01184-0).
